# A shape-memory and spiral light-emitting device for precise multisite stimulation of nerve bundles

**DOI:** 10.1038/s41467-019-10418-3

**Published:** 2019-06-26

**Authors:** Hao Zheng, Zhitao Zhang, Su Jiang, Biao Yan, Xiang Shi, Yuanting Xie, Xu Huang, Zeyang Yu, Huizhu Liu, Shijun Weng, Arto Nurmikko, Yuqiu Zhang, Huisheng Peng, Wendong Xu, Jiayi Zhang

**Affiliations:** 1 State Key Laboratory of Medical Neurobiology and MOE Frontier Center for Brain Science, Institutes of Brain Science, Department of Hand Surgery, National Clinical Research Center for Aging and Medicine, Huashan Hospital, Fudan University, 200433 Shanghai, China; 2 State Key Laboratory of Molecular Engineering of Polymers, Department of Macromolecular Science, and Laboratory of Advanced Materials, Fudan University, 200438 Shanghai, China; 3 School of Engineering, Brown University, Providence, RI 02912 USA

**Keywords:** Optogenetics, Translational research

## Abstract

We previously demonstrated that for long-term spastic limb paralysis, transferring the seventh cervical nerve (C7) from the nonparalyzed side to the paralyzed side results in increase of 17.7 in Fugl-Meyer score. One strategy for further improvement in voluntary arm movement is selective activation of five target muscles innervated by C7 during recovery process. In this study, we develop an implantable multisite optogenetic stimulation device (MOSD) based on shape-memory polymer. Two-site stimulation of sciatic nerve bundles by MOSD induces precise extension or flexion movements of the ankle joint, while eight-site stimulation of C7 nerve bundles induce selective limb movement. Long-term implant of MOSD to mice with severed and anastomosed C7 nerve is proven to be both safe and effective. Our work opens up the possibility for multisite nerve bundle stimulation to induce highly-selective activations of limb muscles, which could inspire further applications in neurosurgery and neuroscience research.

## Abstract (web summary)

Optogenetic stimulation of damaged peripheral nerves has advantages over electrical stimulation but it’s limited to single-site stimulation. Here the authors develop a spiral-shaped LED implant for precise optogenetic stimulation of peripheral nerve bundles at multiple sites and use it to induce distinct limb movements in mice.

## Introduction

**Fig. 1:**
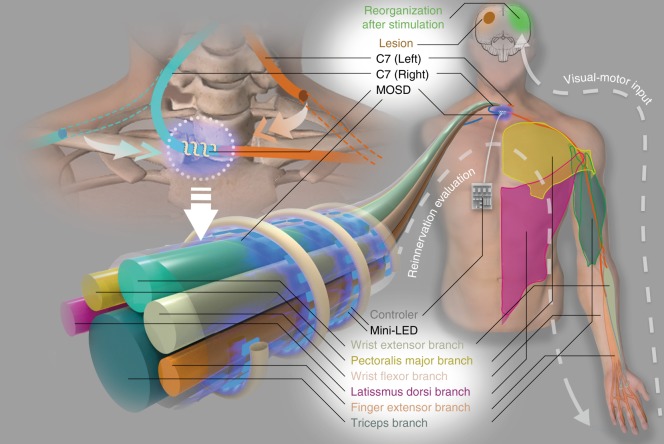
A cartoon of multisite optogenetic stimulation device for therapeutic neuroprosthetics. C7 (~1 cm in diameter in humans) comprises the sensory, pectoralis major, wrist extensor, latissimus dorsi, finger extensor, and triceps branches. In the C7 transfer surgery, C7 on the paralyzed side will be severed and anastomosed directly to the cut end on the nonparalyzed side using microsurgical suturing. The multisite optogenetic stimulation device is comprised of multiple mini-LEDs of 320 μm × 240 μm in physical size and 160 μm × 240 μm in illumination area

Spastic unilateral arm paralysis caused by damage to the cerebral hemisphere from stroke, traumatic brain injury, or cerebral palsy results in long-term disability in patients, severely affects their quality of life, and has become a global social burden^1,2^. We previously developed a surgical procedure for contralateral seventh cervical nerve (C7) transfer from the nonparalyzed side to the paralyzed side^3^, after which patients demonstrated improved motor function and reduced spasticity in the paralyzed arm over 12 months. To further improve the functional recovery of the upper limb, rehabilitation strategies are to be developed post C7 transfer surgery. Recent studies suggest that patterned neuromodulation improves motor control after spinal cord injury^4–6^. In addition, enhancement of selective reinnervated peripheral nerve input to the brain as well as visual-motor feedback to the patient to promote brain reorganization is critical for improvement in functional recovery^7–15^. C7 nerve bundles contain different branches from proximal to distal, including the latissimus dorsi branch and the pectoralis major branch innervating the shoulder muscles, the triceps branch innervating the elbow, the wrist extensor and flexor branch innervating the wrist, and the finger extensor branch innervating the wrist and hand muscles^16–18^ (Fig. 1). Therefore, we aim to develop techniques for selective stimulation of C7 nerves in this study.

Due to the spread of electrical currents in the nerve, electrical stimulation of peripheral nerve bundles has relatively poor spatial resolution. Served as a clinical tool, nerve cuff electrode is suitable for single-site stimulation only^19^. Transverse intrafascicular electrodes accommodate multisite stimulation^20^. However, the regenerated C7 nerve fibers are often too fragile for these penetrating electrodes^3^. Recent development of multi-electrode softening cuffs enabled selective fascicular stimulation in sciatic nerve^21^. However, the number of activated muscles is limited to two.

Optogenetics enabled manipulating activities of genetically targeted cells and has been widely used in the central nervous system^22^. Peripheral nerve modulation using optogenetics is recently considered as emerging technology that can potentially relieve clinical conditions^23^. Optogenetic stimulation of peripheral nerve bundles recruits motor units more efficiently than electrical stimulation^24^. To date, optogenetic devices are single-site optic fiber-based nerve cuffs and mini-LEDs, illuminating the entire nerve bundle^25,26^. Utilizing the retrograde transfection of two distinct optogenetic proteins in peripheral nerve allows opposing muscle activations in the sciatic nerve^27^. However, multisite optogenetic selectivity is limited by the number of genetically tagged types of nerve fibers as well as the number of available optogenetic proteins that have distinct excitation spectra.

We propose a selective multisite optogenetic stimulation device (MOSD) comprised of multiple mini-LEDs for selective stimulation of nerve bundles by spatially separating stimulation sites. Cuff-like architecture serves as backbone of the device, providing full coverage of the entire nerve bundle circumference. Shape-memory polymer^28,29^ provides the mechanical flexibility and stability in shaping the device to adapt to nerve bundles with different sizes (Fig. 1). Polymer^28,29^ with high mechanical stability wraps around nerve bundles. Monte Carlo simulations suggest a 200-µm spatial resolution of one mini-LED. We first implant the MOSD onto sciatic nerve to selectively elicited flexion and extension movements in the ankle joint. MOSD also elicits shoulder adduction, wrist flexion, as well as elbow, wrist and finger extension when implanted onto mice C7. Moreover, the MOSD is implanted onto severed and anastomosed C7 nerve of free-moving mice for 3–8 weeks, proven to be functional and bio-compatible in long term. Our device may open up the possibility of multisite stimulation in nerve bundles to improve rehabilitation after C7 transfer surgery and could also be expanded into multiple fields in neurosurgery and neuroscience research.

## Results

### Fabrication of MOSD

C7 nerve root is ~1 cm in diameter in human being, with about six nerve bundles. To target individual nerve bundles, we chose mini-LEDs with power efficiency 31.2% at 2.9V^30^ (Supplementary Fig. 1a) and the light-emitting area of each mini-LED is 0.24 mm × 0.16 mm. At luminous intensity of 63219 cd m^−2^, the current efficiency is 3.35 cd A^−1,^^30^ (Supplementary Fig. 1b). We measured the luminescence power of LED driven by continuous 2.5 V over the course of 15 h. The fraction change of luminescence power is <2% (Supplementary Fig. 1c), indicating that mini-LEDs we chose could be utilized for long-term nerve stimulation. We also turned on mini-LED at a cycle of 10 s, 1 min and 1 h, respectively and measured the luminescence change (Supplementary Fig. 1d–f), these results indicated that warm-up period is not necessary during stimulation. The MOSD comprises a spiral shape-memory fiber to serve as a backbone and multiple mini-LEDs. Polyurethane fiber is a heat-induced shape-memory polymer and has been extensively used as a clinical material for fillers and brackets because of its shape-memory property^31–33^, bio-compatibility^34–38^, and mechanical compatibility with meniscus and parenchyma tissue^39^. The original shape of the fiber is straight. When heated to 120 °C for 10 min and cooled down to room temperature, the fiber was fixed in a form (1st fixation). After being heated to 120 °C for 10 min for the second time, the fiber returned to the original form (1st recovery). Supplementary Fig. 2 showed the three cycles of fixation-recovery, showing the shape-memory feature of polyurethane fibers. In addition, the polyurethane fiber can be fabricated into different shapes, as demonstrated in Supplementary Fig. 3.

**Fig. 2:**
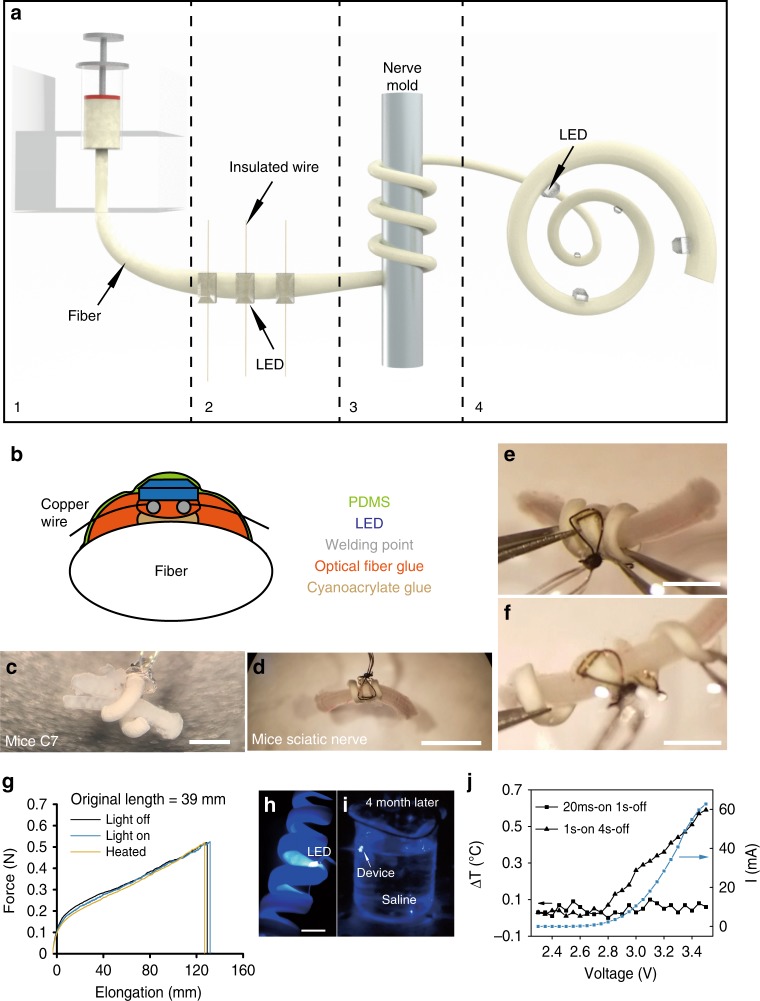
Fabrication and performance of the multisite optogenetic stimulation device (MOSD). **a** Flow chart for MOSD fabrication. (1) Production of a polyurethane fiber; (2) attachment of mini-LEDs to the fiber; (3) shaping of the fiber into a spiral shape; (4) final device. **b** Schematic diagram of device encapsulation. **c**, **d** Images of the MOSD wrapped around nerves with different diameters. **c** Mouse C7 nerve. **d** Mouse sciatic nerve. **e** An image of the MOSD at its original length fixed onto the mouse sciatic nerve. **f** An image of the elongated MOSD in **e**. **g** Mechanical properties of the polyurethane fiber under light or heat. LED was turned on at 2.5 V, 20.7 µA (0.0012 mW). The device was kept at 50 °C in the heated condition (green line). The original length of the MOSD is 39 mm. **h** The MOSD with mini-LEDs turned on in saline. **i** The MOSD with mini-LEDs turned on after 4 months continuously submerged in saline. **j** I–V curve as well as temperature changes at different input voltages for the mini-LED. Voltage applied to the mini-LED varied from 2.3 to 3.5 V (0.00003 mW–33.1 mW). The stimulation patterns for applied voltages are 20 msec-on/2 sec-off and 1 sec-on/4 sec-off. Scale bars are 400 µm (**c**), 2 mm (**d**), and 1 mm (**e**, **f**)

The diameter of the polyurethane fiber was controlled by a spinneret (Fig. 2a, stage 1). Mini-LEDs were first mounted onto the polyurethane fiber with cyanoacrylate and epoxy resin adhesive (Fig. 2a, stage 2). The angle and distance between mini-LEDs were customized according to the size of the target nerve (Fig. 2a, stage 2 and Supplementary Fig. 4a–d). For a rat optic nerve with a 500-μm diameter and 1.6-mm circumference, the distance between mini-LEDs for a 3 mini-LED MOSD was ~500 μm. The mini-LEDs were insulated with polydimethylsiloxane (PDMS), which is optically transparent, insulating, and waterproof (Fig. 2b). Polyurethane fibers with mini-LEDs were then rolled onto a metal rod into a spiral shape (Fig. 2a, stage 3). The diameter of the spiral was set by the nerve mold (Fig. 2a, stage 3). After being heated to 120 °C for 10 min and cooled down to room temperature for 10 min, the fiber was fixed in a spiral form (Fig. 2a, stage 4). MOSDs with different physical sizes were designed to work with peripheral nerves with different diameters (Fig. 2c–d).

The MOSDs demonstrated favorable ductility and exhibited linear elongation when forces were applied longitudinally and remained the same when LED was turned on at 2.5 V, 20.7 µA or heated up to 50 °C (Fig. 2e–g, Supplementary Movie 1). Moreover, the mechanical property of the MOSDs was retained for up to 10 months (Supplementary Movie 2). To test the encapsulation of the MOSD, we soaked the device in saline continuously for over 4 months; after this period, the mini-LEDs were successfully turned on (Fig. 2h–i). The heating of local tissue is a concern for chronically implanted optogenetic devices. We measured the temperature change at the surface of an un-encapsulated LED using Optris Infrared Thermometers. The temperature change was not detectable at 2.5 V, 20.7 µA after being on for up to 4 h (Supplementary Fig. 5a). Differential scanning calorimetry showed that the epoxy resin as well as the silicon elastomer used to encapsulate the MOSD are stable at 40–240 °C (Supplementary Fig. 6). We also measured the heat generated using the stimulation parameters in our experiments (20 msec-on/1 sec-off and 1 sec-on/4 sec-off) using Optris Infrared Thermometers (Fig. 2j, Supplementary Figs. 1 and 5b). The temperature increase was <1 °C, which does not apparently cause nerve damage^40^ or activation^41,42^. We fabricated MOSD using platinum wires for long-term implant experiments, taking into consideration for biocompatibility (Supplementary Fig. 7).

### MOSD 2-site stimulation of mice sciatic nerves

**Fig. 3:**
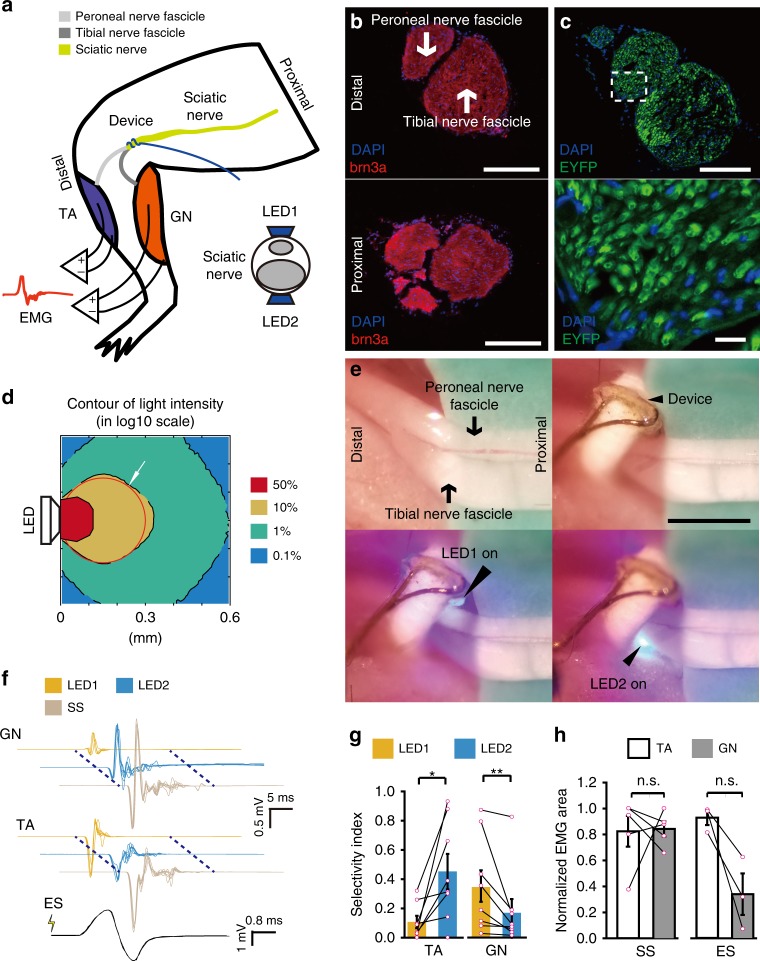
MOSD stimulation of the sciatic nerve in *Thy1*-*ChR2* mice. **a** Schematic diagram of the MOSD attached to the distal side of a sciatic nerve. Stimulation was provided at 0.2 Hz (turned on for 20 msec-on/5 sec-off). Electromyography was performed on the tibialis anterior muscle (TA) and gastrocnemius muscle (GN). **b** Cross section of the sciatic nerve at the location of the MOSD (upper panel) and 4 mm from the location of the MOSD (lower panel). Red staining is brn3a, and blue staining is DAPI. **c** Transverse section of the sciatic nerve. Green staining is ChR2-EYFP, and blue staining is DAPI. **d** Monte-Carlo simulation of mini-LED light intensity across and around the mouse sciatic nerve. The red circle indicates the sciatic nerve in adult mice with a 300-μm diameter. **e** Mini-LED1 and 2 of the MOSD positioned on the distal side of the sciatic nerve to stimulate peroneal and tibial nerve fascicles at 0.026 mW (20 msec on-2sec off), respectively. **f** Representative electromyogram recorded from the TA and GN by different mini-LEDs of MOSD (20. 4 mW power, calculated light intensity at the distal end was 134.8 mW mm^−2^, 20 msec-on/2 sec-off), single-site optogenetic stimulation (81.6 mW power, calculated light intensity at the distal end was 539.2 mW mm^−2^, 20 msec-on/2 sec-off) and electrical stimulation in mice. The electrical stimulation pulse was 0.6 mA for 0.2 msec. **g**, **h** Normalized myoelectric area of the TA and GN in response to different mini-LED stimulations (*n* = 8 mice, 1.31 mW–20.4 mW, 20 msec-on/2 sec-off, TA: Paired *t-*test, GN: Wilcoxon Signed Rank Test), single-site optogenetic stimulation (*n* = 5 mice, paired t test) and electrical stimulation (*n* = 3 mice, 4.55 mW, 20 msec-on/2 sec-off, Paired *t-*test). SS: Single-site optogenetic stimulation; ES: electrical stimulation. Data are presented as mean ± s.e.m.; *P < 0.05, **P < 0.01. Scale bars are 100 µm (**b**, **c** (top)), 10 µm (**c** (bottom)) and 200 µm (**e**)

For proof-of-principle demonstration of selectivity in MOSD, we first conducted 2-site stimulation of mice sciatic nerve using MOSD with 2 mini-LEDs. We previously reported the expression of Channelrhodopsin (ChR2) in the sciatic nerves of *Thy1*-*ChR2* mice^43^. The sciatic nerves of mice branch into the tibial and peroneal nerves, innervating the gastrocnemius muscle (GN) and tibialis anterior muscle (TA) (Fig. 3a). Immunohistochemistry staining revealed the tibial and peroneal nerve branches in the distal and proximal sciatic nerve (Fig. 3b). In *Thy1*-*ChR2* mice, we tested the selective stimulation of tibial and peroneal nerve branches using MOSD. ChR2 was uniformly expressed in the sciatic nerve (Fig. 3c, Supplementary Fig. 8a, b). We performed a Monte-Carlo simulation to examine the propagation of blue light in the sciatic nerve^44–47^. Blue light (peak: 470 nm, 420–520 nm) with intensities greater than 50% of the LED intensity occupied 29.7% of the area at the cross section of the sciatic nerve (Fig. 3d, Supplementary Fig. 8c). For each light emmitance (in mW), we calculated the light intensity at the mini-LED light-emitting surface, middle, and far end of the cross section of the mice sciatic nerve using Monte-Carlo simulation (Supplementary Tables 1 and 2).

The mouse was lightly anesthetized with isoflurane, and the device was carefully placed onto the distal side of sciatic nerve, with mini-LED1 aligned with the peroneal nerve and mini-LED2 aligned with the tibial nerve (Fig. 3e). The mini-LEDs were turned on at ~2.0 V and reached 20.4 mW at 3.3 V (Supplementary Fig. 4e). Given that the active area of mini-LED2 is 160 μm × 240 μm and the diameter of sciatic nerve is 300 μm, we calculated the light intensity at the edge of the nerve using Monte Carlo simulation (see caption of Fig. 3f).

To test whether mini-LED1 and mini-LED2 activate the peroneal and tibial nerves independently, we recorded electromyography (EMG) signals from the GN and TA while optogenetically stimulating the sciatic nerve using the MOSD. The area under the EMG curve indicated the level of muscle activation. Figure 3f–g shows that the EMG area of the GN was significantly larger when mini-LED2 was turned on. Conversely, the EMG area of the TA was larger when mini-LED1 was turned on. All mice showed differential preference in activated muscle when different mini-LEDs were turned on. The area under the EMG curve increased as the light intensity from the MOSD increased (Supplementary Fig. 8d), indicating that the MOSD can recruit more nerve fibers by increasing light intensity. To compare MOSD stimulation with single-site optogenetic stimulation, we provided single-site optogenetic stimulation to the sciatic nerve and found that GN and TA muscles were non-selectively activated (Fig. 3f, h, Supplementary Fig. 9a). We also applied electrical stimulation directly onto the sciatic nerve. In most cases, the EMG area of GN was larger than that of TA (Fig. 3h, Supplementary Fig. 9b). Therefore, unlike MOSD stimulation, both single-site optogenetic stimulation and electrical stimulation lack the selectivity in GN and TA muscles. These results suggested that the MOSD delivers optogenetic stimulation to different branches of the nerve bundle to selectively activate the GN and TA.

**Fig. 4:**
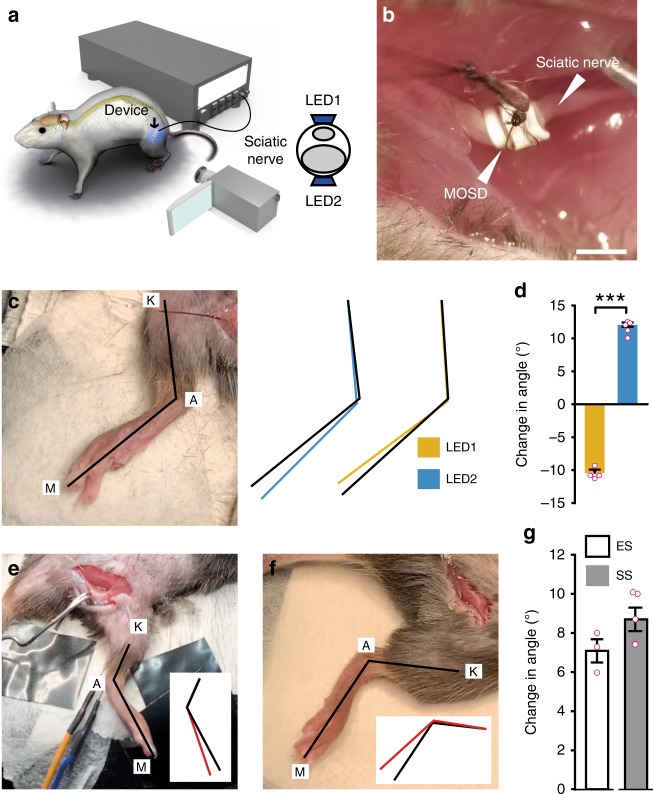
MOSD induced selective extension and flexion in the ankle joint. **a** Schematic diagram of *Thy1*-*ChR2* mice implanted with the MOSD. **b** An image of the MOSD firmly wrapped around the sciatic nerve. **c** The knee joint (K), ankle (A), and metatarsal head (M) were labeled in an anesthetized mouse. Black lines represented the original position of the K-A-M. Blue and Yellow lines represented the positions of K-A-M in response to mini-LED1 and mini-LED2. **d** Average change in the angle of the ankle joint in response to mini-LED1 and mini-LED2 (light turned on at 20. 4 mW for 20 msec with 2-sec intervals, calculated light intensity at the mini-LED: 888 mW mm^−2^; in the middle: 398 mW mm^−2^; at the far end: 134 mW mm^−2^; *n* = 5 mice, Paired t test; ***P < 0.001). **e** Representative image of the position of the leg before electrical stimulation (0.5–0.7 mA, 0.2 msec). Black line in the inset represented the original position of K-A-M. Red line in the inset represented the position of K-A-M after electrical stimulation. **f** Representative image of the position of the leg before the single-site optogenetic stimulation (81.6 mW, 20 msec-on/2 sec-off). Black line in the inset represented the original position of K-A-M. Red line in the inset represented the position of K-A-M after electrical stimulation. **g** Average change in the angle of the ankle joint in response to electrical stimulation (*n* = 3 mice) and single-site optogenetic stimulation (*n* = 5 mice, 81.6 mW). Data are presented as mean ± s.e.m. Extension was defined as positive angle; flexion was defined as negative angle. ES: Electrical stimulation; SS: single-site optogenetic stimulation. Scale bar is 1 mm (**b**)

We further tested whether selective stimulation of the sciatic nerve using the MOSD generates flexion and extension movements in the ankle joint (Fig. 4a). The MOSD was implanted on the distal side of the sciatic nerve in *Thy1*-*ChR2*-*EYFP* mice (Fig. 4b). Prior to the stimulation experiment, the lower limb stretched naturally, and the knee joint (K), ankle (A) and metatarsal head (M) were identified (Fig. 4c). We defined an extension movement as an increase in the K-A-M angle and a flexion movement as a decrease in the K-A-M angle. Turning on mini-LED1 induced flexion in the ankle joint, whereas turning on mini-LED2 alone induced extension (Fig. 4d, Supplementary Movie 3). 37.5% of the mice did not show movement selectivity. When stimulated with 0.5–0.7 mA electrical current for 0.2 msec (Fig. 4e, Supplementary Movie 4), extension in the ankle joint was induced. We also made a MOSD with 4-mini-LEDs (Fig. 4f, inset) and turning on all four mini-LEDs for single-site optogenetic stimulation (Fig. 4f, Supplementary Movie 5). Flexion in the ankle joint was detected. These results demonstrated selective control of flexion and extension movements in the ankle joint by MOSD stimulation of the sciatic nerve. On the other hand, single-site optogenetic stimulation and electrical stimulation induced non-selective movements in the ankle joint.

### MOSD 8-site stimulation of mice C7 nerve

**Fig. 5:**
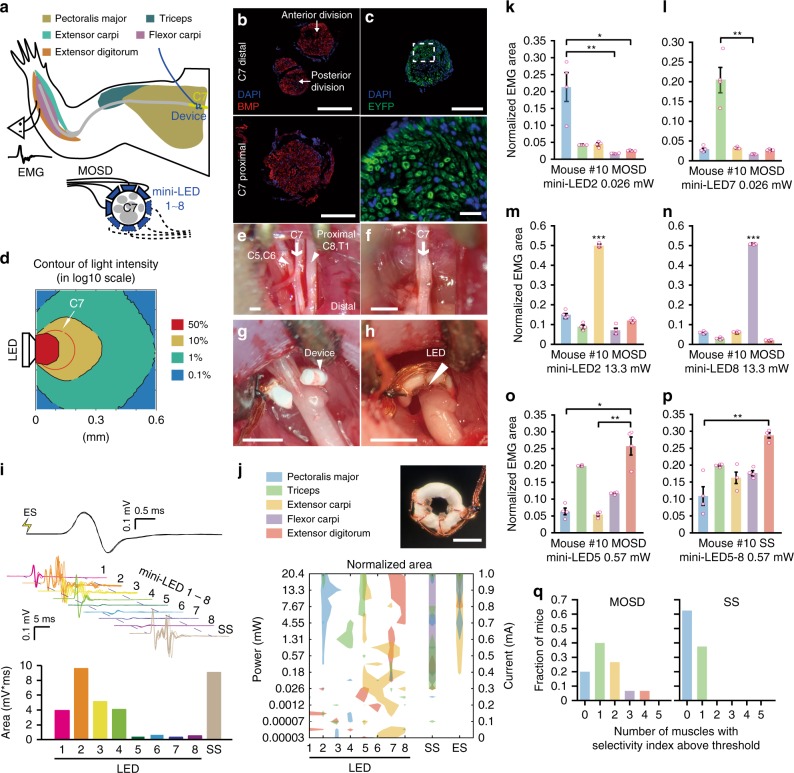
MOSD stimulation of C7 nerve bundle in *Thy1*-*ChR2*-*EYFP* mice. **a** Schematic of experimental set-up. MOSDs were implanted on the proximal side of C7 nerve bundle and electromyography was recorded. **b** Immunofluorescence staining of C7 nerve at distal side (upper) and proximal side (lower). Red, BMP. Blue, DAPI. **c** Expression of EYFP in C7 nerve of *Thy1*-*ChR2*-*EYFP* mice. Green, EYFP. Blue, DAPI. **d** Monte-Carlo simulation of mini-LED light intensity across and around C7 nerve. **e**–**h** Illustration of MOSD implantation process on C7 nerve. **e** Identification of C5–8 and T1 nerves. **f** excise C5–6, 8, and T1 nerves. **g** MOSD implanted on the proximal side of C7 nerve. **h** MOSD was turned 180°. **i** Representative electromyogram from Triceps by different mini-LEDs (20.4 mW, 20 msec-on/2 sec-off, calculated light intensity at the mini-LED: 888 mW mm^−2^; in the middle: 602 mW mm^−2^; at the far end: 267 mW mm^−2^) of MOSD, single-site optogenetic (SS) (81.6 mW, 20 msec-on/2 sec-off), and electrical stimulation (ES) (0.6 mA, 0.2 msec-on/1 sec-off) in one mouse. Myoelectric area was presented below. **j** MOSD (top right) with mini-LED (bottom) representative response pattern of Pectoralis major (PM), Triceps (Tr), Extensor carpi (EC), Flexor carpi (FC), and Extensor digitorum (ED) by different mini-LEDs of MOSD, SS and ES in one mouse. Left Y axis: mini-LED light power. Right Y axis: current used for ES. **k**–**o** Representive mouse showed selectivity for PM, Tr, EC, FC, and ED by MOSD (*n* = 4 trials. **k**, **l**, **o**- Friedman’s ANOVA on Ranks with Tukey post hoc; **m**, **n**: One way ANOVA with Tukey post hoc, *P < 0.05, **P < 0.01, ***P < 0.001). **p** The same mouse showed selectivity in only one muscle by SS. **P < 0.01, *n* = 4 trials, Friedman’s ANOVA on Ranks with Tukey post hoc. **q** Summarized distribution of the fraction of mice with numbers of muscles which showed selectivity (*n* = 15 mice for MOSD, *n* = 8 mice for SS). Data are presented as mean ± s.e.m. Scale bars are 100 µm (**b**, **c** (top)), 10 µm (**c** (bottom)) and 400 µm (**e**–**h**, **j** (top right))

The C7 nerve bundle of mice innervates the Pectoralis major muscle (PM), Latissmus dorsi muscle (LD), Triceps muscle (Tr), Extensor carpi muscle (EC), Flexor carpi muscle (FC), and Extensor digitorum muscle (ED) (Fig. 5a). We manufactured MOSD with four mini-LEDs that covered half of C7 nerve and turned the device 180° to cover the entire C7 during the stimulation experiment. Immunohistochemistry staining revealed that axons were densely packed in both the distal and proximal side of C7 nerve (Fig. 5b). In *Thy1*-*ChR2* mice, ChR2 was uniformly expressed (Fig. 5c). We noted that C7 nerve is smaller than the sciatic nerve. Previous study shows that the scattering coefficient of rat spinal cord is 2.9–3.5 mm^−1^ at 400–1000 nm^48^, similar to that of rat optic nerve. We therefore used the optical parameters of mice sciatic nerve for mice C7 nerve. Monte Carlo simulation showed that the light intensity in the entire C7 was above 10% of LED intensity (Fig. 5d, Supplementary Table 3).

Figure 5e showed the exposed C7 nerve. To ensure that all the recorded muscle responses were from C7, we severed and excised C5, C6, C8, and T1 prior to the stimulation (Fig. 5f). The mice lied on their back during EMG recording. To avoid turning over the mice during recording, EMG signals from latissimus dorsi muscle were not recorded. An MOSD device with four mini-LEDs was implanted onto C7 (Fig. 5g, marked as LED 1–4). Each individual LED was turned on one by one, driven by a range of voltages (2.3–3.3 V). After EMG of the C7 innervating muscles was recorded, the MOSD was turned 180° (Fig. 5h, marked as LED 5–8) and EMG was recorded thereafter. In some mice, not all five muscles had responses, and data from these mice were not included into the analysis below.

Figure 5f showed a representative EMG recording from Triceps muscle. The myoelectric area for Pectoralis major muscle with LED 2 on was selectively larger than that for the rest LEDs (Fig. 5i). To obtain an overview of the MOSD stimulation on all five muscles, we plotted LEDs 1–8 with different light intensities in one mouse in the same figure (Fig. 5j). Each colored territory indicated that at the corresponding LED/light intensity, the normalized myoelectric area from a specific muscle is above 95% of the peak myoelectric area. Many of the colored territory were labeled with one color, suggesting that one muscle was selectively activated. The LED/light intensity corresponding to single-colored territory would thus be chosen as selective stimulation conditions. When LEDs 1–4 were turned on, namely single-site optogenetic stimulation, EMG recording showed that the myoelectric areas of ED, PM, Tr, EC, and FC muscles were all above 95% threshold (Fig. 5j). Likewise, ED, PM, Tr and EC muscles were activated when C7 was electrically stimulated (Fig. 5j). Moreover, in both single-site optogenetic stimulation and electrical stimulation, the relative ratio of activation levels in the five muscles remained the same at different light intensities or input current (Supplementary Fig. 10).

We calculated the selectivity index ((Area_max_−Area_second_max_)/(Area_max_ + Area_second_max_), see Methods for details) for each illumination condition (Supplementary Fig. 11a). Figure 5k–p showed that, while different illumination conditions delivered by MOSD could result in different preferences in the activation of EC, FC, PM, ED, and Tr muscles in the same mouse, single-site optogenetic stimulation could only yield activation in one muscle at most.

We defined a threshold (mean + 2 s.d., Supplementary Fig. 11b) for selectivity index to quantitatively compare MOSD optogenetic stimulation of C7 to single-site optogenetic stimulation as well as electrical stimulation. 80% mice had one or multiple muscles above threshold in MOSD stimulation, whereas 37.5% mice had only one muscle above threshold in single-site optogenetic stimulation (Fig. 5q, Supplementary Fig. 11c). These results suggested that MOSD stimulation of C7 nerve provided an effective means for selective activation of PM, Tr, EC, WF, and ED muscles.

We also implanted MOSD with three mini-LEDs onto optic nerve of rats and conducted electrophysiology recordings in V1, which demonstrated the selective 3-site optogenetic stimulation of optic nerves (Supplementary Figs. 12–14, Supplementary Tables 4–5).

**Fig. 6:**
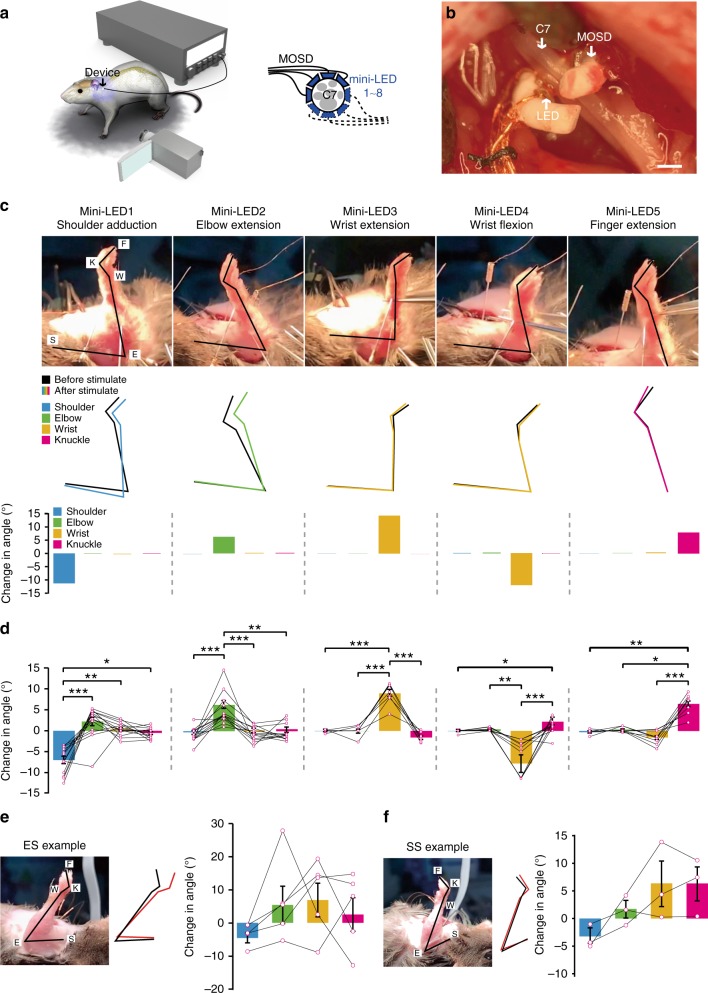
MOSD induced distinct upper limb movement in *Thy1*-*ChR2* mice. **a** Schematic of C7 nerve implanted with MOSD and experimental set-up. **b** Image of MOSD wrapped around C7 nerve bundle. **c** Representative images of upper limb movement elicited by different mini-LEDs of MOSD (20.4 mW, 20 msec-on/2 sec-off, calculated light intensity at the mini-LED: 888 mW mm^−2^; in the middle: 602 mW mm^−2^; at the far end: 267 mW mm^−2^). F, fingertip. K, knuckle. W, wrist. E, elbow. S, shoulder. Black line represented the position of upper limb before mini-LED stimulation. Colored line represented the position of upper limb after mini-LED stimulation. **d** Joint angle movement of shoulder, elbow, wrist, and knuckle response to mini-LEDs (shoulder adduction, *n* = 14 mice; elbow extension, *n* = 14 mice; wrist extension, *n* = 7 mice; wrist flexion, n = 12 mice; finger extension, *n* = 10 mice) in 1.31–20.4 mW (20 msec-on/2 sec-off) (shoulder adduction, elbow extension, wrist flexion, finger extension: Friedman’s RM ANOVA on Ranks with Tukey post hoc; wrist extension: One way RM ANOVA with Tukey post hoc. *P < 0.05, **P < 0.01, ***P < 0.001). **e** Representative images of upper limb movement elicited by electrical stimulation (ES) (0.8 mA, 0.2 msec-on/1 sec-off). Joint angle movement of shoulder, elbow, wrist, knuckle under electrical stimulation (*n* = 5 mice). **f** Representative images of upper limb movement by single-site optogenetic stimulation (SS) (81.6 mW, 20 msec-on/2 sec-off). Joint angle movement of shoulder, elbow, wrist, knuckle in single-site optogenentic stimulation (middle right) and MOSD stimulation (right). Extension was defined as positive angle; flexion was defined as negative angle (*n* = 3 mice). Data are presented as mean ± s.e.m. Scale bar is 200 µm (**b**)

We next explored the locomotion of mice upper limb when C7 nerve was selectively stimulated by MOSD. Similar to the setup in Fig. 5, we implanted 4-mini-LED MOSD onto C7 and turned it 180° for 8-LED stimulation (Fig. 6a, b). A high-speed camcorder was used to record the movement of the upper limb when different LEDs were turned on. Figure 6c showed that LEDs 1–5 elicited shoulder adduction, elbow extension, wrist extension, wrist flexion as well as finger extension in the upper limb respectively in one mouse (Supplementary Movie 6). The locomotion from the shoulder, elbow, wrist, and knuckle joints were selective when one LED was turned on (Fig. 6d). We would like to note that in 38.8% mice, different mini-LEDs elicited similar locomotion patterns. This may be due to the different C7-innervation patterns in different mice. Electrical stimulation of C7 nerve elicited a more complex pattern of locomotion in the upper limb comparing to single mini-LED stimulation (Fig. 6e, Supplementary Movie 7). Figure 6f showed that single-site optogenetic stimulation by turning on mini-LEDs 1–4 also elicited complex movement patterns in different joints (Supplementary Movie 8). Together with the results from EMG recordings, MOSD stimulation of C7 nerve was more likely to elicit selective activation of upper limb muscles comparing to electrical stimulation or single-site optogenetic stimulation.

### Long-term biocompatibility of MOSD in mice C7

**Fig. 7:**
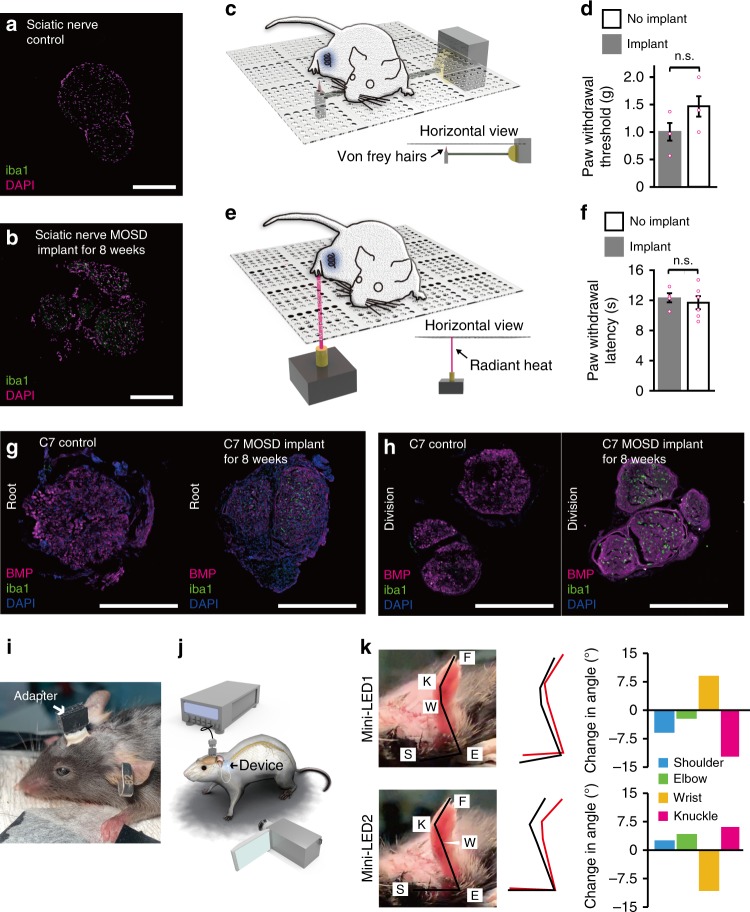
Biocompatibility and effectiveness of MOSD after 8-week implant. **a**, **b** Immunostaining of mice sciatic nerve after 8 weeks MOSD implant. **c**, **d** Withdrawal latency of mice in von Frey test. MOSD were implanted into sciatic nerve for 4 weeks. *n*_implant_ = 4 mice, *n*_no implant_ = 6 mice, Unpaired *t-*test. **e**, **f** Withdrawal latency of mice in Hargreaves test. MOSD were implanted into sciatic nerve for 4 weeks. Sham operation group were taken as control. *n*_implant_ = 4 mice, *n*_no implant_ = 6 mice, Unpaired *t-*test. **g**, **h** Immunostaining of mice C7 nerve after 8 weeks MOSD implant in the root and division end. **i**, **j** Schematics and images of mice with MOSD implant. Adapter for the MOSD implant was fixed onto the skull. **k** Different upper limb movements were elicited by mini-LEDs of MOSD (81.6 mW, calculated light intensity at the distal end was 539.2 mW mm^−2^, 20 msec-on/2 sec-off) 3 weeks after the C7 implant. Black lines represented the position of upper limb before mini-LED stimulation. Red lines represented the position of upper limb after mini-LED stimulation. Data are presented as mean ± s.e.m. Scale bars are 200 µm (**a**, **b**, **g**, **h**)

We then tested the biocompatibility and multisite-stimulation feature of the implanted MOSD. Figure 7a, b showed the mice sciatic nerve 8 weeks after MOSD implant. There was a slight increase in the number of microglial cells. Von Frey and Hargreaves’ test showed that no pain-related behavior was observed in mice whose sciatic nerve was implanted with MOSD for 4 weeks (Fig. 7c–f).

Figure 7g–h showed the existence of regenerated axons at both the root and division sides of the C7 nerve 8 week after the C7 sever and anastomosis surgery and implantation of MOSD afterwards. Note that C5–6, C8, and T1 were also severed and excised in these C7 MOSD implanted mice. Similar to mice sciatic nerve, there is a slight increase in the number of microglial cells (Fig. 7g–h). We also fabricated a multichannel head-stage and implanted onto the mice skull for MOSD stimulation after long-term implant (Fig. 7i–j). The regenerated axons in C7 nerve reach the hand muscle 3 weeks after C7 surgery and MOSD implant. Two mini-LEDs of the MOSD elicited different movements in shoulder, elbow, wrist, and knuckle joints of the mice upper limb, demonstrating that MOSD elicited selective activation of the regenerated C7 nerve (Fig. 7k, Supplementary Movie 9).

We further assessed the utility of the MOSD for long-term implantation in freely moving rats (Supplementary Fig. 15a). The MOSD was implanted onto the optic nerve after intravitreal injection of the AAV-hSyn-ChR2-mCherry virus (Supplementary Fig. 15b). A flexible printed circuit adapter for electrical connections to mini-LEDs was mounted onto the rat’s skull (Supplementary Fig. 15c). One week after the implant surgery, the rat was able to move around freely with the MOSD (Supplementary Fig. 15d). Optic nerve was intact 6-weeks after the MOSD implant (Supplementary Fig. 15e). We designed an associative learning experiment to test whether the animal was able to respond to optogenetic activation via the MOSD by pairing MOSD stimulation with water reward. Prior to the behavioral experiment, the animal was placed under water restriction and injected with 5–6 μL glutamate receptor antagonist cocktail, which took effect within 30 min, in both eyes to block the intrinsic light response (Supplementary Fig. 14). The mini-LEDs were turned on for 2 sec with simultaneous administration of 0.05 mL water (Supplementary Fig. 15f). The associative training consisted of 10 trials per day and lasted for 3 consecutive days. Correct licking behavior was defined as the animal licking for water within 5 sec after the light was turned on. The success rate (correct licking/all licking) of water-licking behavior significantly increased on day 3 (Supplementary Fig. 15g), indicating that the association between MOSD-induced optic nerve activation and water reward was established in freely moving rats.

Different mini-LEDs on MOSD activate subgroups of nerve fibers and induce different activation patterns in V1. We then conducted two-choice Y-Maze experiments to address the question of whether different mini-LEDs in MOSD can elicit different behaviors in rats. We trained the MOSD-implanted rats to associate mini-LED1 with left arm while mini-LED2 with right arm. If the rats chose the correct arm, they got two sucrose pellets as reward. Otherwise, the reward was omitted (Supplementary Fig. 15h). After training for about two weeks, when the rats were cued with mini-LED1, there was a higher chance that they went to the left arm. Meanwhile, the rats preferred to choose the right arm when they were cued with mini-LED2 (Supplementary Fig. 15i). These data suggest that different LEDs in MOSD elicited different behaviors in free-moving rats.

## Discussion

In this study, we developed an implantable device for multi-site optogenetic stimulation in peripheral nerves. The shape-memory material enabled the adaptation of MOSD to nerves with different diameters. We first conducted Monte Carlo simulation to validate the local distribution of visible light in the peripheral nerves. We then demonstrated that 2-site MOSD stimulation of mice sciatic nerve induced flexion and extension in the ankle joint. More excitingly, 8-site MOSD stimulation in mice C7 selectively induced shoulder adduction, wrist flexion, as well as elbow, wrist, and finger extension. When chronically implanted, MOSD elicited differential movements by stimulating regenerated C7.

To justify the necessity for the multisite feature of MOSD stimulation, we showed that electrical stimulation in C7 induced EMG responses in shoulder, elbow, wrist, and finger muscles. However, tuning the stimulation current did not change the pattern of responses in the muscles. Results from single-site optogenetic stimulation were similar as those in electrical stimulation. Alternatively, one could implant multiple electrodes or LEDs onto nerve terminals that innervate selective muscles^49^. Many nerve terminal branches are just below the arch and deep into the muscle belly^50^, so dissection of multiple nerve terminals is surgically challenging and causes damage to the surrounding tissue and blood supply to the muscles. Using MOSD, we were able to program the on/off patterns of four mini-LEDs in C7, aiming at maximization of selective muscle activation.

Nerve bundles are consisted of multiple nerve fibers innervating different muscles. Previous studies showed that distribution of nerve fibers in some nerve bundles may be topographically arranged according to the targets before nerve branches^51–53^. Detailed microanatomical studies revealed the fascicular topography of the brachial plexus^16,54^, indicating some level of somatotopic organization in C7. Results from our Monte Carlo simulation showed that the minimum activation depth for MOSD is 100 µm, suggesting that optogenetic stimulation may be localized to different fascicles that distributed close to the surface of C7; when light intensity was turned up, the activation depth increases accordingly. With MOSD implanted onto C7, although not in all mice, different mini-LEDs preferentially activated shoulder, elbow, wrist and hand muscles, respectively. Due to the limitation in animal usage as well as the difference between currently available mice model and human patients, the detailed mechanism remained to be explored. However, our current study provided a horizon for improvement in rehabilitation after C7 transfer surgery.

Safety and biocompatibility issues are important for long-term implants of MOSD. Local heating effect from turning on the mini-LED could be damaging to nerve tissue, especially regenerated fibers that tend to be more fragile than normal ones. Our experiments showed that polyurethane fibers have stable mechanical properties under light or heat. Nerve tissue staining indicated that no significant inflammation was found after implanted into mice sciatic nerve and C7 nerve for 8 weeks. No pain-related behavior was observed 4 weeks after MOSD implant onto sciatic nerve, demonstrating that MOSD implant does not cause adverse sensory responses.

Recent literature suggested the optogenetic promotion of axon regeneration, including corticospinal tract^55,56^ and retinal axons in mice^57^. However, optogenetics is not immediately ready yet for clinical translation. Nevertheless, there are a few active clinical trials that utilize optogenetics to treat retinitis pigmentosa (NCT02556736, phase 1&2; NCT03326336, phase 1 &2). In summary, despite a few limitations of the current version of our multisite optogenetic stimulation device including adaptation for long-term implant and translational prospects, our device offers the potential role in a way of selective peripheral neuromodulation, as well as future prospects in selective stimulation of other peripheral nerves.

## Methods

### Material preparation

Preparation of polyurethane fiber: polyurethane was first dissolved in dimethyl formamide (0.6 g mL^−1^) at 80 °C for 1 h with strong mechanical stirring. Subsequently, polyurethane solution was transferred to a 20-mL single-hole (diameter = 300 μm) spinneret and then injected into a dimethyl formamide/water coagulation bath (v/v, 6/4) to obtain the polyurethane fiber. The fiber was immersed in the coagulation bath for 5 min and then transferred to a distilled water coagulation bath for another 5 min. After drying at 50 °C for 2 h, the polyurethane fiber was obtained.

### MOSD fabrication and characterization

Insulated copper or platinum wire (diameter = 50 μm) was welded onto the two leads of a mini-LED (wavelength = 450 nm; backplane = 240 × 320 μm^2^; luminous area = 140 × 220 μm^2^; thickness = 115 μm; C450TR2432-S2400, CREE, Inc., USA), and brushed epoxy resin adhesive (TRA-BOND F123 BIPAX, THORLABS, Inc., USA) was used to cover the bipolar metal sheet. The mini-LEDs were attached to the same side of the polyurethane fiber with the luminous surface facing up. The distance between the mini-LEDs was determined by the size of the target nerve as well as the number of mini-LEDs. The copper or platinum wire was wound around the polyurethane fiber and fixed with epoxy resin adhesive. The polyurethane fiber with mini-LEDs was wound around a metal rod into a spiral shape (steel rod, diameter determined by the size of the target nerve) and fixed with clips. The device was placed on a hot plate (KW-4AH, CHEMAT Technology, lnc., USA) at 150 °C for 15 min and cooled to room temperature in a ventilated environment for 1 h. Finally, PDMS (Silicon Elastomer, SYLGARD, DOW CORNING, Inc., USA) was brushed onto the surface of the mini-LEDs to a thickness of ~50 μm and heated on a hotplate at 60 °C for 30 min.

For acute implantation, we connected the copper or platinum wires with a 2-mm male head pin (2-mm spacing cable adapter). For long-term implantation, we bound the copper or platinum wires and connected them to the flexible printed circuit adapter (0.5 mm). The welding points on the adapter were covered with epoxy resin (AB Adhesive, MICHEL, Inc., CHN).

The mini-LED was driven and recorded by Keithly 2400 Source Meter. The luminance was tested by a spectroradiometer (Photo Research PR680) and the luminous power was measured by an optical power and energy meter (THORLABS PM100D). The luminance stability for 15 h and on/off cycles were measured by an optical fiber photometric (Admesy Asteria).

The LED’s temperature and thermal mapping pictures were collected by Optris Infrared Thermometers. 20 msec-on/1 sec-off and 1 sec-on/4 sec-off pulse signals were selected to simulate LEDs for the tests of the temperature changes under different bias voltages. The local temperatures and thermal mappings were recorded after working for 3 min. The long-time temperature changes were evaluated under 2.5 V for 4 h.

The mechanical properties of light on, off and heated states were tested by electric universal testing machine (Hengyi, Shanghai). The driven voltage of LEDs was 2.5 V. Heat was applied to fiber by an infrared heating lamp. The local temperature of fiber was about 50 ℃, measured by a handheld infrared radiation thermometer.

The shape memory of polyurethane fiber was operated under 100 ℃ for 10 min and fiber was cooled down quickly in the room temperature. It was heated to 100 ℃ for 10 min again to recover the shape. The stability of polymer components used in the device assembly was evaluated by differential scanning calorimetry (TA, Q2000).

### Monte-Carlo simulation of photon transport

The simulation of light spread by mini-LED stimulation in the nerves and surrounding tissue was performed in two steps: the generation of incident light distribution from mini-LEDs and the simulation of photon transport in the tissue.

The active area of the mini-LED has dimensions of 0.22 mm (length) × 0.14 mm (width), on which we assumed uniform intensity of light emission. To obtain the angular distribution of the incident light, we first extracted and interpolated (‘pchip’) the angular emission pattern of the mini-LEDs in the width dimension with a custom MATLAB script based on datasheets (company reference).

The distribution of optical stimulation in biological tissue is widely modeled through the Monte-Carlo simulation of photon transport in a scattering medium. For a complete review, please refer to the work by Wang et al. ^58^. Here, we adapted the implementation by Ozden et al.^59^ and Stujenske et al.^60^ for applications with mini-LEDs.

The volume of the simulated tissue was at least a 1 mm^3^ cube (4 mm × 4 mm × 1 mm for axial scenarios), modeled with the same isotropic optical properties as the optic nerve or the sciatic nerve. The nerves were positioned just below the surface, while the mini-LED was juxtaposed above. Refraction was considered at the interface between the mini-LED and tissue (*n* = 1.40); a continuous boundary (no refraction or reflection) was implemented at the interface of the nerve and the rest of the simulation volume.

The diameters of nerves are 0.5 mm for the rat optic nerve, 0.3 mm for the mouse sciatic nerve, and 0.2 mm for the mouse C7 spinal nerve bundle. For both nerves, the refractive index is 1.40, the absorption coefficient is 0.5 mm^−1^, and the anisotropy is 0.9. The reduced scattering coefficient of the optic nerve is 2.59 mm^−1^ and that of the sciatic nerve is 1.56 mm^−1^. The lower value in scattering coefficient of the sciatic nerve would lead to more pronounced light distribution along the direction of incidence.

In short, we launched >10^**7**^ photons following the spatial distribution of the incident LED light and tracked the trajectory of each photon within the volume of simulation. Then we computed the normalized power density (mW mm^−3^) and light intensity distribution (transmittance, mW mm^−2^) in this volume, at corresponding input power levels (mW).

### Animals

Animal care and experiments were performed in accordance with the National Institutes of Health Guide for the Care and Use of Laboratory Animals and were approved by the Animal Care and Use Committee of Shanghai Medical College of Fudan University. Wild-type (Wistar) rats were obtained from the Shanghai Laboratory Animal Center, CAS (Shanghai, China). *Thy1*-*ChR2*-*EYFP* mice (https://www.jax.org/strain/012350) were purchased from the Jackson Laboratory. All animals were housed at 22 °C, with 12-h light/dark cycles. Room humidity was controlled at 50%. All experiments were conducted during the light cycle. The weights of water-restricted rats were maintained at 85% of their ad libitum body weight.

### Intravitreal injection and analysis of retinal projections

Anesthetized rats (4 weeks old) were intravitreally injected with 8–9 μL AAV-hSyn-ChR2-mCherry (OBIO Y4460 Inc., USA) in the left eye. Rats were allowed to recover for 2 weeks before the next experiment.

Rats were anesthetized with chloral hydrate (0.5 g kg^−1^) and injected intravitreally with 100 nL CTB-488 (4.17 µg µL^−1^) and CTB-555 (0.91 µg µL^−1^) (Molecular Probes, Invitrogen, Inc., USA, C22842, C22843) by NanoJectII (Drummond Scientific Company, USA) at 69 nL/injection, with 1–2 injections each in the nasal (CTB-555), temporal (CTB-488), dorsal (CTB-555), and ventral (CTB-488) directions. After 36–48 h, rats were perfused transcardially with 0.01 phosphate buffer (PB) followed by 4% paraformaldehyde (PFA, Sigma, USA, P6148) in PB. Brains were removed and stored in 4% PFA overnight, whereas retinas were stored in 4% PFA for 2–3 h. The brains were coronally sectioned in a vibratome (Leica Inc., BRD, VT1000 S, Germany) to obtain lateral geniculate nucleus slices (100-μm thick). Four slices from each mouse with the largest dorsal lateral geniculate nucleus and ventral lateral geniculate nucleus areas were collected and stained with DAPI (Invitrogen, Inc., USA, 1:3000) for further analysis. Lateral geniculate nuclei were visualized and photographed using a microscope (Olympus, Inc., JPN, BX51) in autoexposure mode with a 3% spot at the brightest fluorescence area. Six images with both green, red, and blue channels were taken to capture the whole lateral geniculate nucleus. These images were merged using Automerge in Olympus BX51 software.

### Optogenetic virus injection

Anesthetized rats (4 weeks old) were intravitreally injected with 8–9 μL AAV-hSyn-ChR2-mCherry (OBIO Y4460 Inc., USA) into the left eye, which was covered with a layer of erythromycin eye ointment. Rats were allowed to recover for two weeks.

### MOSD implant and optogenetic stimulation on sciatic nerve

*Thy1*-*ChR2* mice were anesthetized with 1.5% isoflurane. After shaving and skin disinfection were performed, the vastus lateralis muscle and biceps femoris muscle were blunt dissected to expose the sciatic nerve. The device was implanted at the distal end of the sciatic nerve with 2 mini-LEDs facing the common peroneal nerve fascicle and tibial nerve fascicle. A needle electrode was used to perform electromyography on the GN and TA, and data were collected using an AM1800 amplifier (Datawave Technology, Inc., USA) and AXON DIGIDATA 1440 A digital analog converter (AXON Inc., BMU). Mini-LED1 was first turned on for 20 msec with 4-sec intervals, and mini-LED2 was turned on following the same procedure. Different voltages (2.3–3.3 V, 0.00003–20. 4 mW, 20 msec-on/2 sec-off) were randomly applied, and the procedure was repeated four times (Supplementary Table 6). For electrical stimulation of sciatic nerve, electrical stimulation probe was wrapped around sciatic nerve. Electrical stimulatory pulse (duration: 0.2 msec, frequency: 1 Hz) was delivered by Medtronic KeyPoint Portable EMG device. The Amplitude of stimulatory pulse was from 0.1 mA to 1 mA every 0.1 mA and evoked EMG was recorded by Medtronic KeyPoint Portable EMG device. Single-site optogenetic stimulation was presented using a 4-mini-LED MOSD device, with all four mini-LEDs turned on.

### MOSD implant and optogenetic stimulation on C7 nerve

Mice were anesthetized with 1.5% isoflurane and the chest and forelimb were shaved. After C5, C6, C8, and T1 were severed and excised, C7 nerve of *Thy1*-*ChR2* mice was exposed and implanted with MOSD. Multisite optogenetic stimulation of C7 nerve was performed by turning on each mini-LED of MOSD from 2.3 V to 3.3 V every 0.5 V (Supplementary Table 6). Single-site optogenetic stimulation of C7 nerve was performed by activating all mini-LEDs of MOSD. The stimulation was repeated six times and electromyography data was recorded by Spike2 and analyzed by Python3. For electrical stimulation, electrical stimulation probe was wrapped around C7 nerve. Electrical stimulatory pulse (duration: 0.2 msec, frequency: 1 Hz) was delivered by Medtronic KeyPoint Portable EMG device. The Amplitude of stimulatory pulse was from 0.1 mA to 1 mA every 0.1 mA and evoked EMG was recorded by Medtronic KeyPoint Portable EMG device.

### In vivo EMG recording in Sciatic and C7 nerve

EMG electrodes were fabricated for muscle recording using acupuncture needles and connected with 0.062-inch female pin connectors by insulated copper wire. Mice were anesthetized with 1.5% isoflurane and the chest and forelimb were shaved. SC or C7 nerve of *Thy1*-*ChR2* mice was exposed. EMG electrodes were implanted into Pectoralis major, Tricep, Extensor carpi, Flexor carpi, and Extensor digitorum muscles respectively. Pin connectors of EMG electrodes were connected to the headstage of Model 1800 microelectrode AC Amplifier (A-M systems). Recordings were amplified by Model 1800 with X1000 signal gain and data was digitized and acquired at 30 kHz using Micro1401–3 and Spike2 software (Cambridge Electronic Design Limited).

Selectivity index of a C7-innervating muscle for each illumination condition was calculated as (Area_max_ − Area_second_max_)/(Area_max_ + Area_second_max_), where Area_max_ is the maximum normalized myoelectric area of each muscle (Pectoralis major, Triceps, Extensor carpi, Flexor carpi, and Extensor digitorum) for each mouse and Area_second_max_ is the second maximum normalized myoelectric area of each muscle. A threshold was defined as mean +2 s.d. (see Supplementary Fig. 15 for details).

### Ankle joint and upper limb movement analysis

The video of limb movement was recorded using IPhone XS Max (Apple Inc. USA), and we got the frame before movement as the pre-frame and the frame with the maximal joint motion as post-frame. In C7 induced upper limb movement experiment, the reference point was marked at shoulder joint, elbow joints, wrist joints, and knuckles, and metatarsal head. In sciatic nerve induced ankle movement experiment, the reference points are marked at knee joint, ankle joint and metatarsal head. The reference point is connected and joint angle data in pre- and post-frame was measured. The change in joint angle was analyzed by comparing the pre-frame and the post-frame.

### MOSD implant in optic nerve and V1 electrophysiology

Rats were anesthetized with 1.5% isoflurane on a stereotaxic setup (RWD, China). A sterilized heating pad (CHEMAT TECHNOLOGY, Inc., USA) was used to maintain body temperature. The posterior part of the left eye was disinfected, and a 2-mm vertical opening was cut 1 mm temporal to the canthus for bulbar conjunctival circumcision. The optic nerve was exposed by blunt separation. We wound the fiber around the optic nerve and fixed the wires to the posterior cartilage with sutures to maintain the relative position and angle between the device and the optic nerve.

After implantation surgery, 9 μL of a visual blocker cocktail (listed below) was intravitreally injected into the rat’s left eye. Erythromycin eye ointment was used for disinfection, and the visual blocker cocktail started to take effect within 30 min. Lidocaine (10 mg mL^−1^ in saline, MP Biomedicals, Inc., USA) was injected subcutaneously under the scalp. After the scalp was cut open, the position of V1 was marked on the skull, and a 2 mm × 2 mm craniotomy was performed. The dura was carefully removed, and the brain was buffered and warmed using sterile buffered saline (37 °C, 150 mM NaCl, 2.5 mM KCl, 10 mM HEPES, pH 7.4) throughout the experiment. Isoflurane was maintained at 0.5% throughout the recording. A cocktail of glutamatergic receptor blockers (AP4 + AP5 + NBQX at 1:1:1 (mol L^−1^), AP4, Tocris, Inc., UK, (23052–81–5). AP5, Tocris (79055–68–8). NBQX, Tocris (118876–58–7)) was injected intravitreally before the recording. A multichannel electrode (Tungsten Microelectrode Arrays, 16187–364, MicroProbes, Inc., USA) was inserted into V1 at a depth of 200–300 μm. The V1 response was recorded for 2 min after mini-LED stimulation, which consisted of 4 cycles of 1-sec stimulation with 4-sec intervals (Supplementary Table 6). The signals were preamplified (Microelectrode AC Amplifier 1800, A-M Systems, Inc., USA), sampled at 10 kHz, and high-pass filtered at 1 Hz.

Electrophysiology data were analyzed in Spike2 (Cambridge Electronic Design Ltd. Inc., UK). Signals were high-pass filtered at 300 Hz and sorted using Offline Sorter (PLEXON. Inc., USA). We set −4.5 thresholds for the spike at a signal-to-noise ratio of 1.5. The firing rate during visual stimulus and nonvisual stimulus was calculated from sorted spikes.

### Long-term MOSD implant in mice C7

Prepare a MOSD (4–6 cm wire) mounted with four mini-LEDs. Mice were anesthetized with 1.5% isoflurane and the chest and forelimb were shaved. C7 nerve of *Thy1*-*ChR2* mice was exposed. Make an incision in the mouse’s scalp from the midpoint between eyes to the midpoint between ears. Introduce the MOSD from the scalp incision, bypass the neck and shoulders from under the skin, then exit from the skin incision on the chest. After the brachial plexus is exposed, cut off the C5, C6, C8, and T1 nerves, sparing only the C7 nerve. We cut the C7 and re-sew it to simulate the clinical C7 fracture injury. Spiral the device around C7-reconnected, suture the wire to the Pectoralis major, coil the extra length under the skin, and suture the Pectoralis major with the chest skin. The MOSD adapter is fixed to the skull with dental cement (Adhesive Cement System, C&B MetaBond, Inc., Japan). Wait for the dental cement to coagulate completely, then suture up the scalp to avoid exposure of the skull (Fig. 7i). After mice recovered for 3 weeks, we conducted the stimulation experiment. We also fabricated a miniaturized stimulation headstage (Supplementary Fig. 16) for optogenetic stimulation in free-moving mice.

### MOSD implant in rat optic nerve and behavioral test

Rats (6 weeks old, male) with ChR2 expression were anesthetized with chloral hydrate (0.5 g kg^−1^) intraperitoneally. As described above in MOSD implant, the fiber with mini-LEDs was wrapped around the optic nerve, and the wires were fixed to the posterior cartilage with sutures. The wires then went beneath the scalp and were connected to the flexible printed circuit adapter. The rat was fixed on a stereotaxic apparatus, and a longitudinal cut was made to expose the skull. Skull screws were fixed to the skull (lambda + 1.2 mm, lateral ± 2 mm), and the flexible printed circuit adapter was mounted on the skull with dental cement.

After surgery, rats were housed individually and allowed to recover for 1 week. Rats were food- or water-restricted 48 h before the behavioral training and were maintained at 85% of their ad libitum body weight. For water administration behavior, the training comprised 3 sessions across 3 consecutive days. In each session, 10 stimuli were presented. During each stimulus presentation, mini-LEDs were activated for 2 sec at 0.18 mW. Water reward was available for 5 sec and then withdrawn. At the end of each training day, the rats were given additional water to maintain their weight. For 2-choise Y maze behavior, on day 1, the rats were habituated to the environment of by allowing them to explore the Y maze freely for 10 min, with sucrose pellets as reward at the end of the two goal arms. In training sessions, the rats were first cued with mini-LED1 (0.18 mW, 20 msec-on/20 msec-off) or mini-LED2 (0.18 mW, 500 msec-on/500 msec-off) for 10 s, then placed into Y maze to choose the goal arm for sucrose reward (Supplementary Table 6). When the rats were cued with mini-LED1, sucrose pellets were only placed at the end of left arm. So only if they chose the left arm, they can get sucrose reward. When the rats were cued with mini-LED2, the baited arm was right arm. The rats received 10 trails per training day and in each trail, the cue was given randomly. The rats with >70% correct rate for 3 consecutive days were used for analysing the response rate in Supplementary Fig. 15i.

### Immunohistochemistry

Cryosectioned slices (cross sections and longitudinal sections; 14-μm thick) were washed with Tris-buffered saline 5 times (5 min each) and permeabilized in 0.5% Triton-X-100 for 30 min. The slices were then blocked in 10% Donkey serum for 2–3 h at room temperature and incubated with primary antibodies overnight at 4 °C (DsRed (Rabbit, Clontech, Inc., USA (632496), 1:800), Brn3a (Goat, Santa Cruz Biotechnology, Inc., USA (sc-31984), 1:300), GFP (Chicken, Abcam, Inc., UK (Ab13970)), 1:500), iba1 (Rabbit, Wako, Inc., JP (019–19741)), 1:800), BMP (Rat, Abcam, Inc., UK (Ab7349)), 1:800)). The slices were washed again 5 times (5 min each), and secondary antibodies (Jackson ImmunoResearch Laboratories, Inc., USA) were applied to the slices and incubated at room temperature for approximately 2 h in the dark. The slices were washed 3 times (5 min each), stained in a 1:3000 DAPI working solution for 7 min and washed 2 more times. Finally, the slides were mounted and photographed with a confocal microscope.

### Statistical analysis

Statistical analyses were performed in Excel (Microsoft Corporation, Inc., USA) and MATLAB (MathWorks, Inc., USA). Data distributions were tested for normality using the Kolmogorov–Smirnov normality test. For comparison of two groups, data with normal distribution were analyzed using paired t test, if data distributions were not normally distributed, Wilcoxon signed-rank test were performed, except that Fig. 7d, f were analyzed with Student’s *t*-test. For comparison of multiple groups, data with normal distribution were analyzed using one way repeated measures ANOVA followed by Tukey post hoc analysis, if data distributions were not normally distributed, Friedman’s repeated measures ANOVA on Ranks followed by Tukey post hoc comparisons. Two way repeated measures ANOVA was used in analyzing Y maze behavioral data in Supplementary Fig. 15i. *P* value < 0.05 was considered statistically significant. Data are presented as the mean ± s.e.m. A summary of the statistical analysis in the manuscript was shown in Supplementary Table 7.

### Reporting summary

Further information on research design is available in the Nature Research Reporting Summary linked to this article.

## Supplementary information

Supplementary Information

Description of Additional Supplementary Files

Supplementary Movie 1

Supplementary Movie 2

Supplementary Movie 3

Supplementary Movie 4

Supplementary Movie 5

Supplementary Movie 6

Supplementary Movie 7

Supplementary Movie 8

Supplementary Movie 9

Reporting Summary

## Data Availability

The data underlying Figs. 3, 5 and Supplementary Figs. 9–12 are provided in the following 10.12751/g-node.52349d. Rest of the relevant data is available from the corresponding author upon reasonable request.

## Code availability

Computer codes in MATLAB (MathWorks Inc., USA) and Python are available from the corresponding author upon reasonable request.
