# Supplementary material for: A shape-memory and spiral light-emitting device for precise multisite stimulation of nerve bundles
Source: Nat Commun. 2019 Jun 26;10:2790. doi: 10.1038/s41467-019-10418-3 (PMC6594927; doi:10.1038/s41467-019-10418-3)
Supplement: Supplementary file 1 — Supplementary Information [file 41467_2019_10418_MOESM1_ESM.pdf]

Supplementary Information

A Shape-Memory and Spiral Light-Emitting Device for Multisite Precise Stimulation of Nerve Bundles

Hao Zheng[1,4], Zhitao Zhang[2,4], Su Jiang[1,4], Biao Yan[1,4], Xiang Shi[2], Yuanting Xie[1], Xu Huang[1], Zeyang Yu[3], Huizhu Liu[1], Shijun Weng[1], Arto Nurmikko[3], Yuqiu Zhang[1], Huisheng Peng[2,5], Wendong Xu[1,5], Jiayi Zhang[1,5]

[1]State Key Laboratory of Medical Neurobiology and MOE Frontier Center for Brain Science, Institutes of Brain Science, Department of Hand Surgery, National Clinical Research Center for Aging and Medicine, Huashan Hospital, Fudan University, Shanghai 200433, China.

[2]State Key Laboratory of Molecular Engineering of Polymers, Department of Macromolecular Science, and Laboratory of Advanced Materials, Fudan University, Shanghai 200438, China.

[3]School of Engineering, Brown University, Providence, Rhode Island 02912, USA.

[4]These authors contributed equally: Hao Zheng, Zhitao Zhang, Su Jiang, Biao Yan

[5]These authors jointly supervised this work: Jiayi Zhang, Wendong Xu and Huisheng Peng and; E-mail addresses: jiayizhang@fudan.edu.cn, wendongxu@fudan.edu.cn and penghs@fudan.edu.cn.

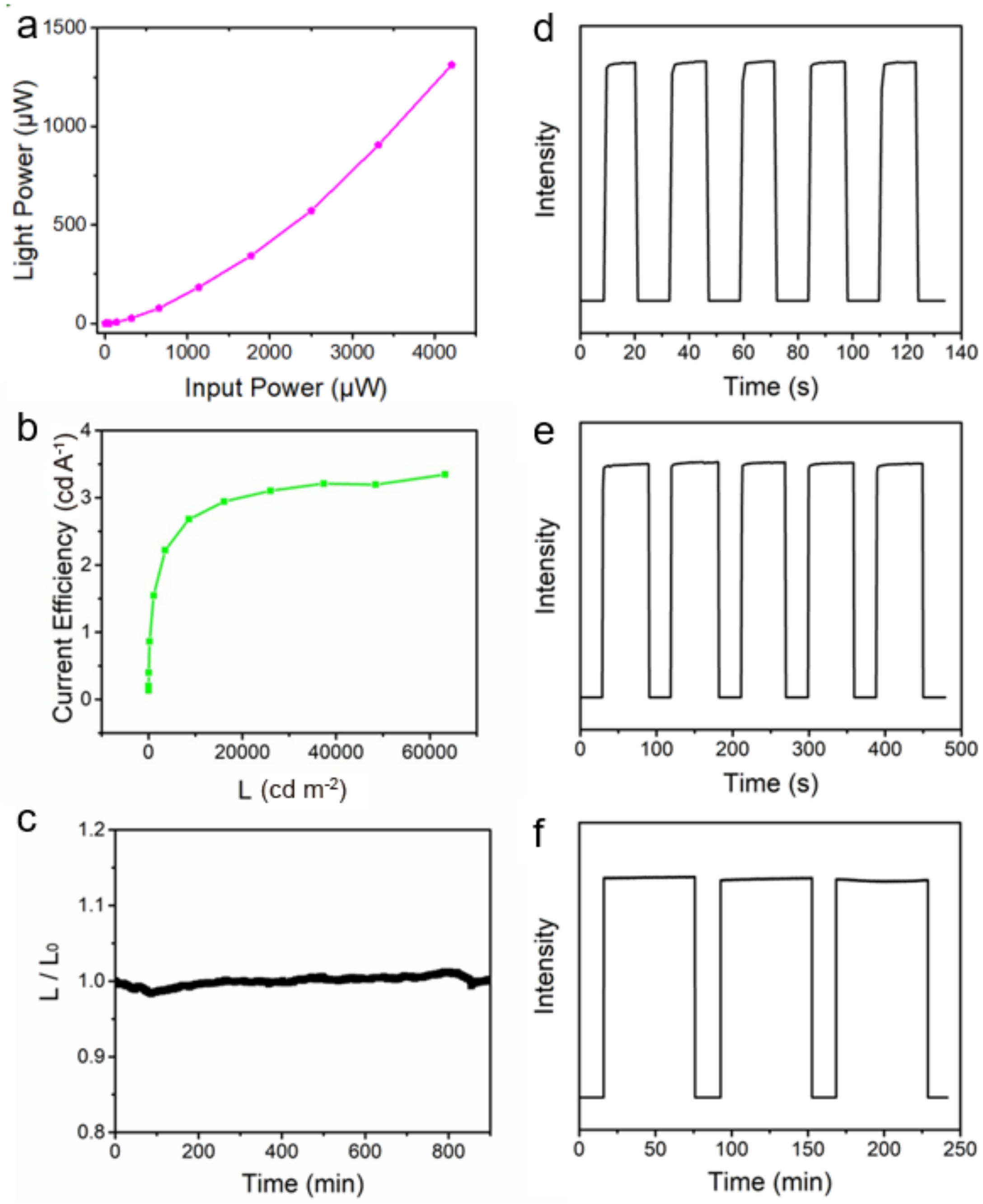

Supplementary Figure 1. Characterization of mini-LED. (**a**) Power efficiency for one mini-LED. Voltages applied to each mini-LED were 2.3 – 2.9 V. (**b**) Current efficiency for one mini-LED. Voltages applied to each mini-LED were 2.3 – 2.9 V. L: luminescence. (**c**) Normalized luminescence of one mini-LED in 15 hr. L/L0: luminescence power normalized

by the value at the beginning of the test (t = 0). (**d-f**) Luminescence of one mini-LED upon ON cycles. The duration of ON cycle is 10 sec, 1 min and 1 hr, respectively.

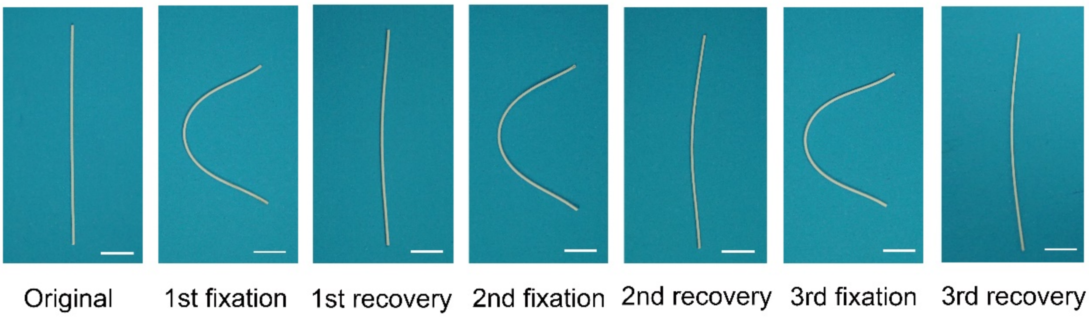

Supplementary Figure 2. Shape-memory feature of polyurethane fibers. One single polyurethane fiber went through the process of 1st fixation – 1st recovery – 2nd fixation – 2nd recovery – 3rd fixation – 3rd recovery. Scale bar: 1 cm.

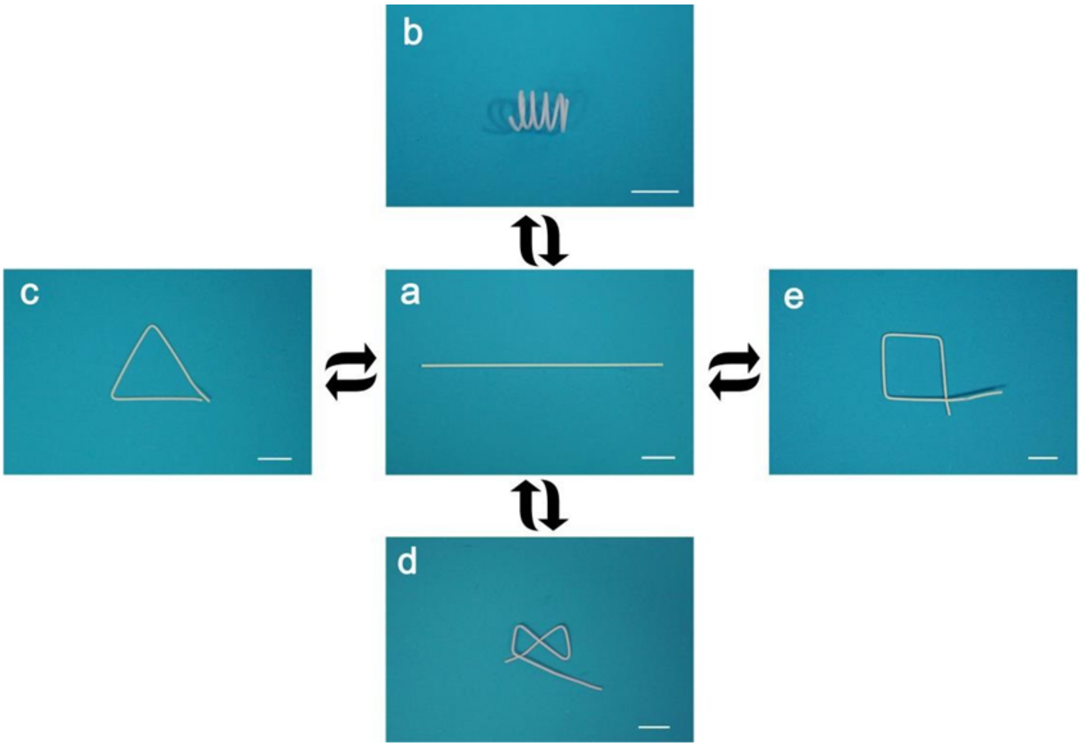

Supplementary Figure 3. Polyurethane fibers were fabricated into different shapes. (**a**) Original shape of the fiber; (**b**) spiral-shaped fiber; (**c**) triangle-shaped fiber; (**d**) knot-shaped fiber; (**e**) squire-shaped fiber. Scale bar : 1 cm.

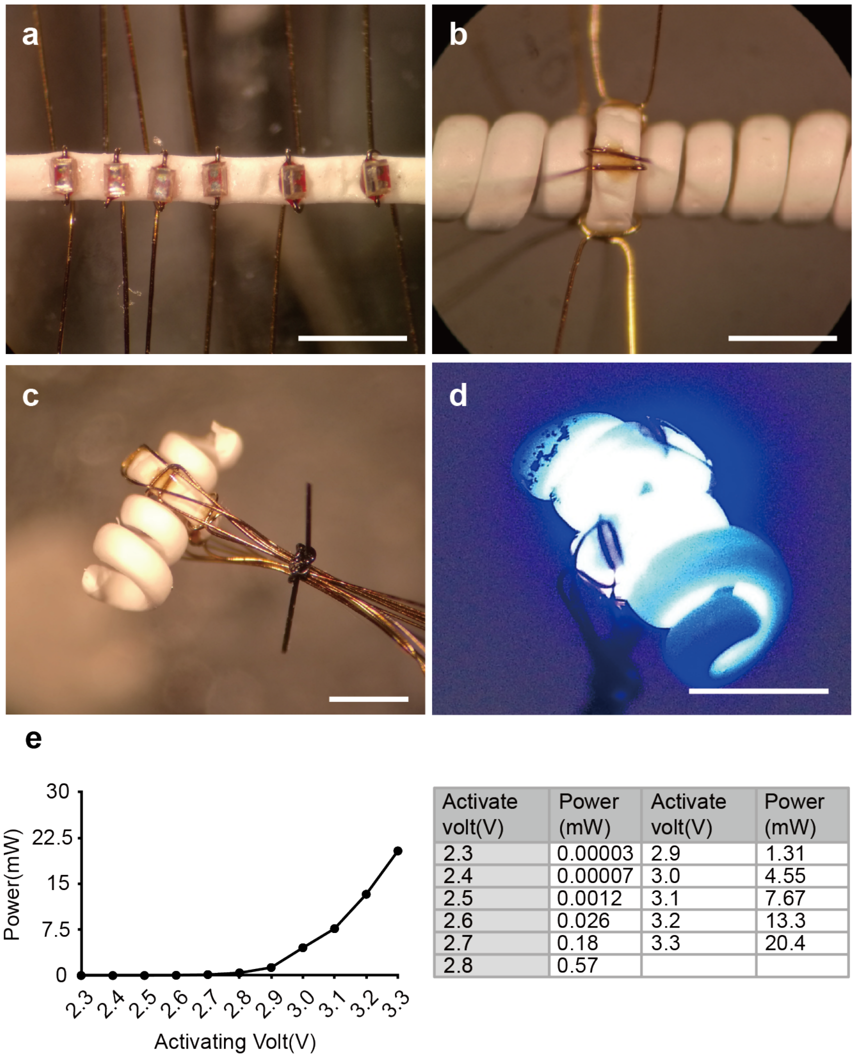

Activate volt(V)	Power (mW)	Activate volt(V)	Power (mW)
2.3	0.00003	2.9	1.31
2.4	0.00007	3.0	4.55
2.5	0.0012	3.1	7.67
2.6	0.026	3.2	13.3
2.7	0.18	3.3	20.4
2.8	0.57		

Supplementary Figure 4. MOSD fabrication. (**a**) Mini-LEDs with insulated copper wires were attached to the fiber. (**b**) The fiber was twined and spiral shaped. (**c**) Restrained copper wire. (**d**) MOSD with mini-LEDs on. (**e**) Light power driven by different voltages. Scale bars are 500 μm (**a-d**).

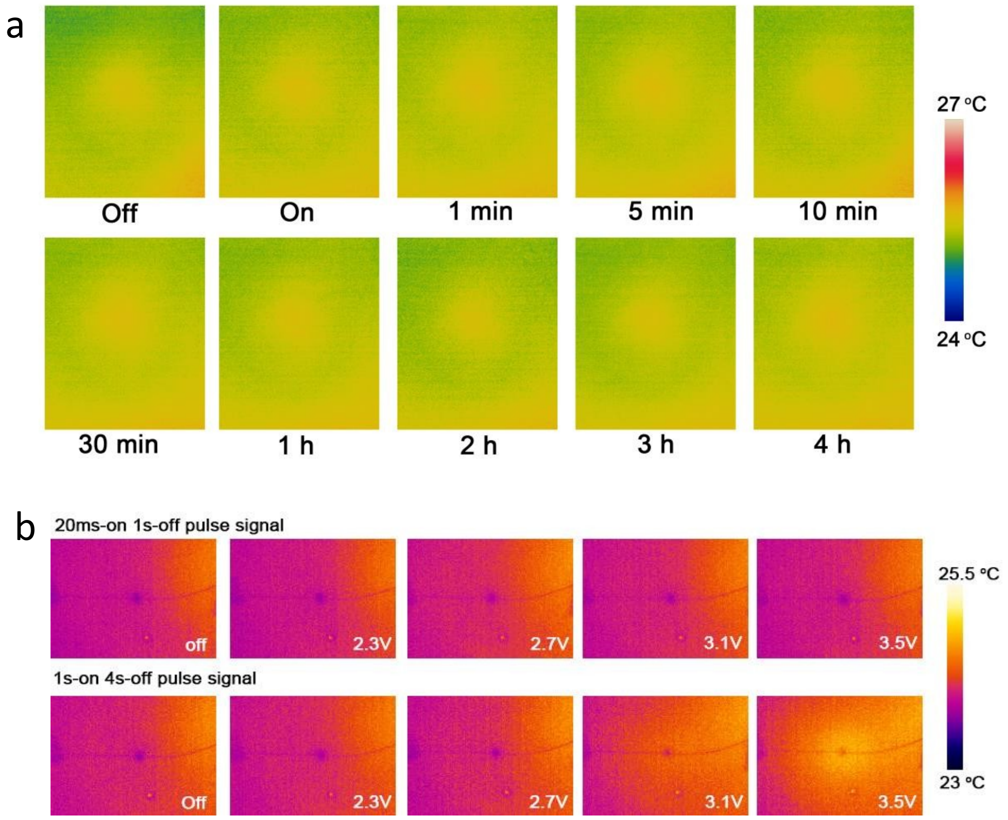

Supplementary Figure 5. Temperature change of one LED. (**a**) Mini-LED was turned on continuously at 2.5 V (0.0012 mW) and the temperature was measured by Optris Infrared Thermometers. (**b**) Mini-LED was turned on from 2.3V to 3.5V (0.00003 mW - 33.1 mW) at both 20 msec-on/1 sec-off and 1 sec-on/4 sec-off modes.

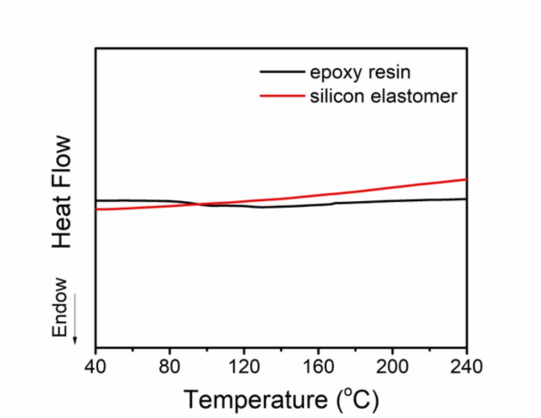

Supplementary Figure 6. Differential scanning calorimetry (DSC) of epoxy resin and silicon elastomer used for MOSD encapsulation.

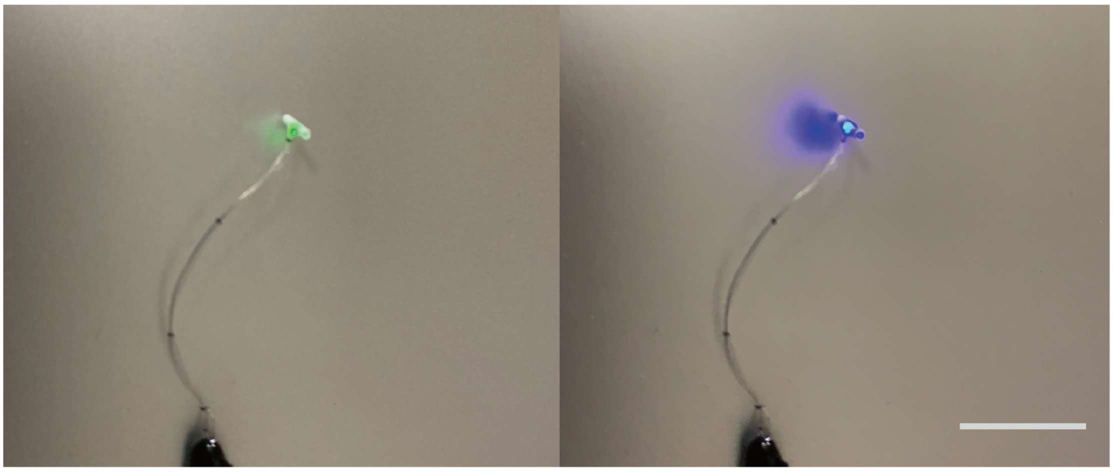

Supplementary Figure 7. MOSD fabricated with platinum wires. Both blue and green mini-LEDs were fabricated onto the same MOSD. Scale bar is 5 mm.

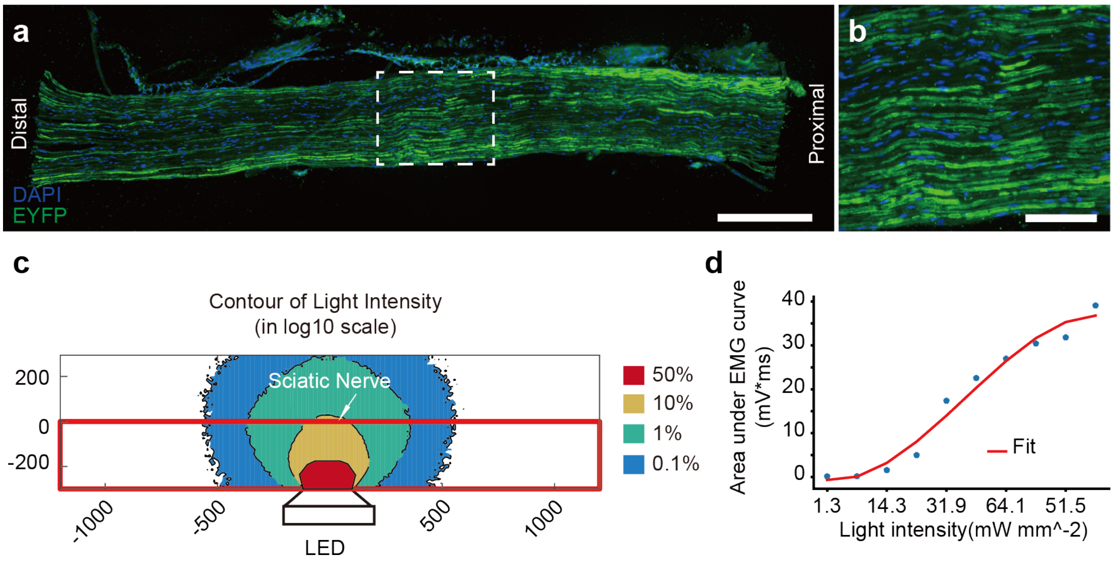

Supplementary Figure 8. Immunofluorescence images of transverse sciatic nerve in Thy1-ChR2-EYFP mice. (**a-b**), Transverse section of sciatic nerve. Green, ChR2-EYFP. Blue, DAPI. (**c**) Monte-Carlo simulation of LED light intensity distribution across and around the mouse sciatic nerve on transverse section. (**d**) EMG areas stimulated by MOSD with different voltages. Scale bars are 300 µm (**a**) and 100 µm (**b**).

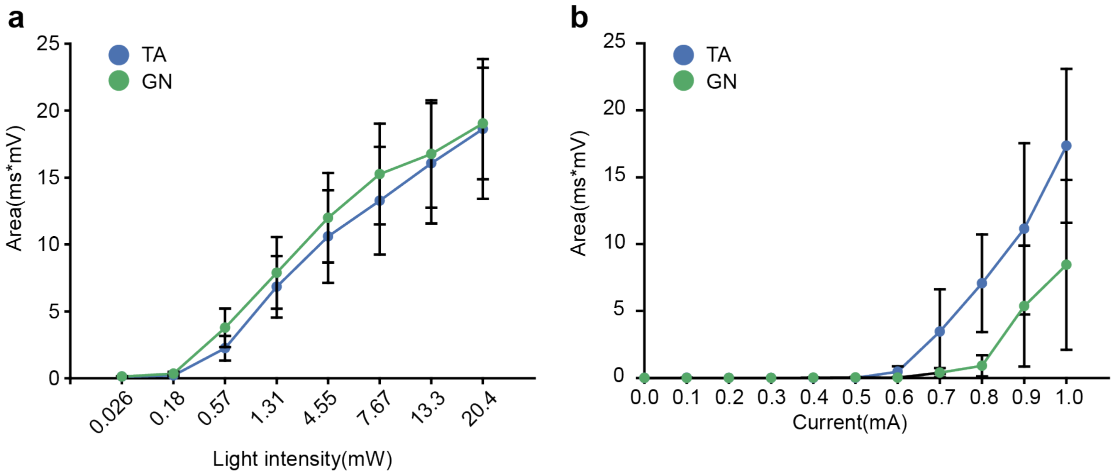

Supplementary Figure 9. EMG responses of GN and TA muscles under electrical and single-site optogenetic stimulation. (**a**) EMG area with single-site optogenetic stimulation (n = 5 mice, 20 msec-on/ 2 sec-off). (**b**) EMG area with electrical stimulation (n = 3 mice, 0.2 msec-on/ 1 sec-off). Data are presented as mean ± s.e.m..

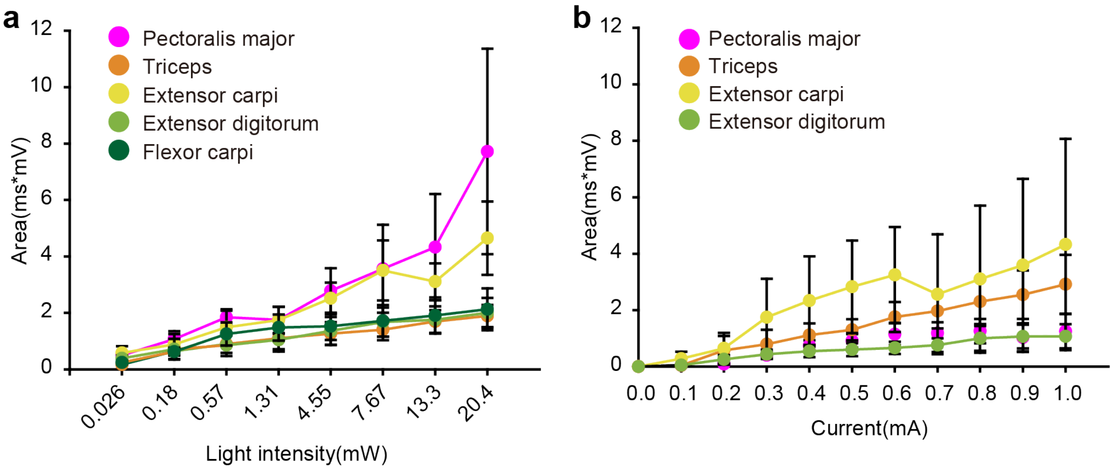

Supplementary Figure 10. EMG responses of PM, Tr, EC, FC and ED muscles under electrical and single-site optogenetic stimulation with different stimulation current. (**a**) EMG area with single-site optogenetic stimulation (n = 7 mice, 20 msec-on/ 2 sec-off). (**b**) EMG area with electrical stimulation (n = 3 mice, 0.2 msec-on/ 1 sec-off). Data are presented as mean ± s.e.m.

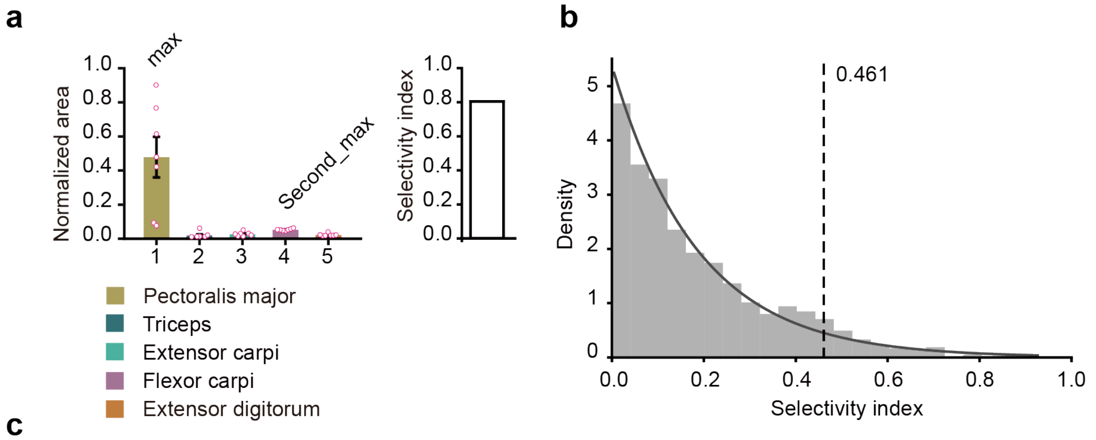

c

mouse index	number of muscles with above-threshold selectivity (MOSD)	number of muscles with above-threshold selectivity (SS)
1	0	0
2	1	1
3	3	0
4	2	0
5	2	0
6	1	1
7	1	0
8	1	0
9	0	1
10	4	0
11	2	0
12	1	0
13	1	0
14	0	0
15	2	0

Supplementary Figure 11. Distribution of selectivity indices and definition of selectivity threshold. (**a**) Example normalized myoelectric area and selectivity index under one illumination condition (the illumination condition from different mouse is 0.00003 mW - 20.4 mW) for one mouse (6 trials, mouse #4 from Supplementary Figure 15 b). (**b**) Histogram of density distribution of selectivity indices of all mice under delivered with MOSD. Selectivity index was defined as ($Area_{max}$ – $Area_{second\_max}$) / ($Area_{max}$ + $Area_{second\_max}$) (See Methods for details). Threshold for selectivity index was 0.46 (mean + 2 s.d.) (1.31 mW). Distribution fitted with exponential function. (**c**) Summary of number of muscles and number of illumination conditions with selectivity indices above threshold for each mouse (0.00003 mW - 20.4 mW).

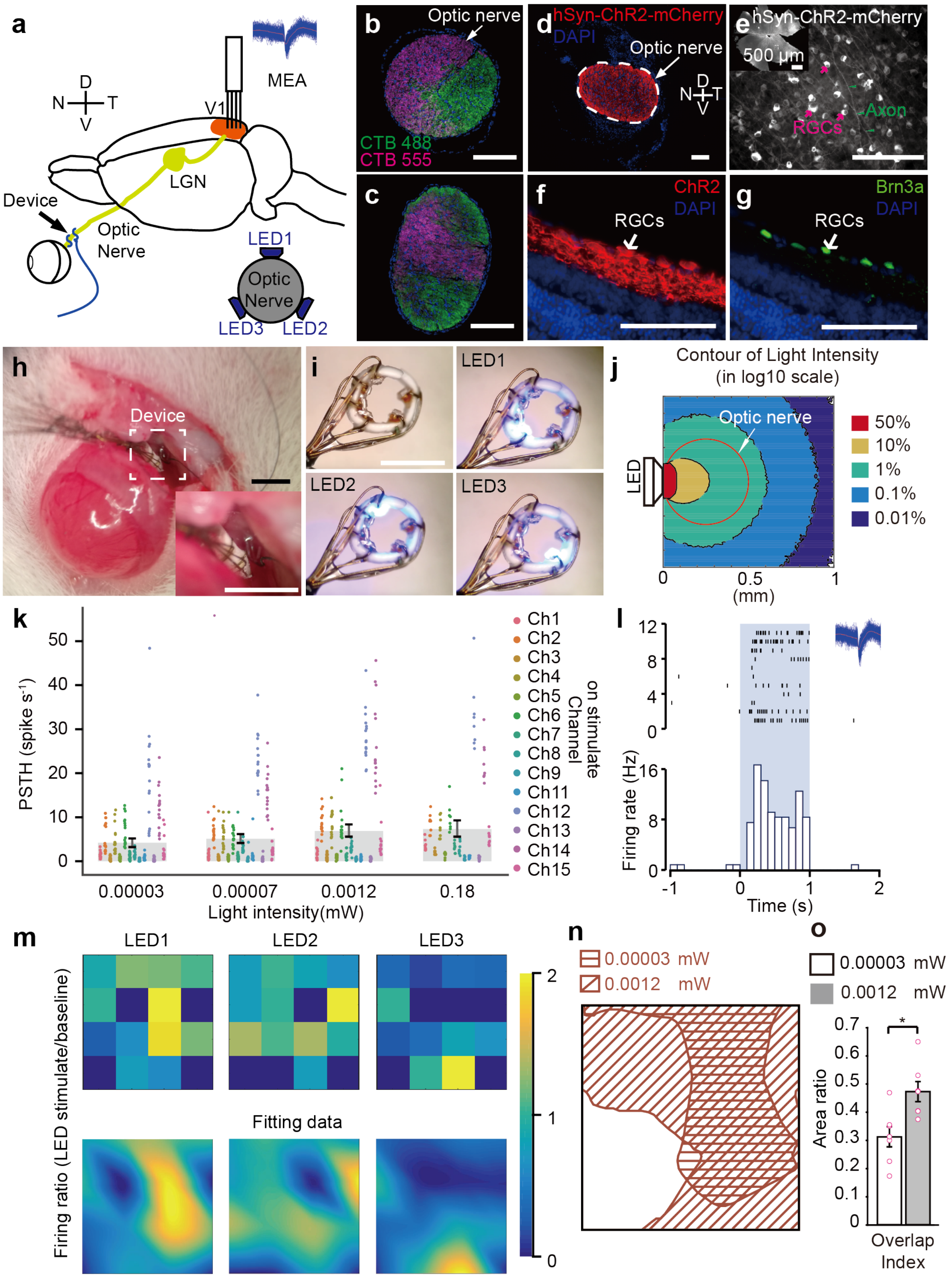

Supplementary Figure 12. Implanted MOSD for retinotopic activation. (**a**) Schematic diagram of the wiring and electrophysiological recording of nerve fiber activation on V1 in rat with intraocular AAV-hSyn-ChR2-mCherry injection. Three mini-LEDs were placed around the optic nerve. MEA: multichannel electrode array, D: dorsal, V: ventral, N: nasal, T:

temporal. (**b-c**) Cross section of the optic nerve 1.5–2 mm from the eyeball. Green, CTB488. Red, CTB555. Blue, DAPI. (**b**) CTB555 was intraocularly injected into the nasal retina, and CTB488 was intraocularly injected into the temporal retina. (c) CTB555 was intraocularly injected into the dorsal retina, and CTB488 was intraocularly injected into the ventral retina. (**d**) ChR2 expression in the optic nerve. Red, ChR2-mCherry. Blue, DAPI. (**e-g**) ChR2 expression in retinal ganglion cells in the retina. Green, brn3a. Blue, DAPI. (**h**) The MOSD implanted on the optic nerve. (**i**) Images of three mini-LEDs turned on individually at 0.0012 mW. (**j**) Monte-Carlo simulation of the LED light intensity distribution across and around the rat optic nerve, indicated by the red circle. Note, an adult rat optic nerve has a diameter of 500 μm, which is larger than that of the mice sciatic nerve (300 μm). (**k**) Peristimulus time histogram (PSTH, spike s^{-1}) of V1 neurons stimulated with different light intensities (turned on for 1 sec-on/4 sec-off at 0.18 mW) by mini-LED1–3 (0.00003 mW - 0.18 mW). (**l**) Local field potential, power spectrum density, and PSTH of V1 neurons in response to MOSD stimulation (turned on for 1 sec-on/4 sec-off at 0.18 mW). (**m**) Heatmap (upper) and Lowess fitting (lower) of the firing ratio (firing rates with the MOSD turned on divided by those with the MOSD turned off) at 0.00003 mW. (**n**) Lowess fitting of the MOSD-activated area (firing ratio > 1) at 0.00003 mW and 0.0012 mW. (**o**) Overlap index of V1 activation area induced by different mini-LEDs at 0.00003 mW and 0.0012 mW (n = 7 mice, Paired t test, $*P < 0.05$.). Data are presented as mean ± s.e.m. Scale bars are 100 μm (**b-g**), 1 mm (**h-i**).

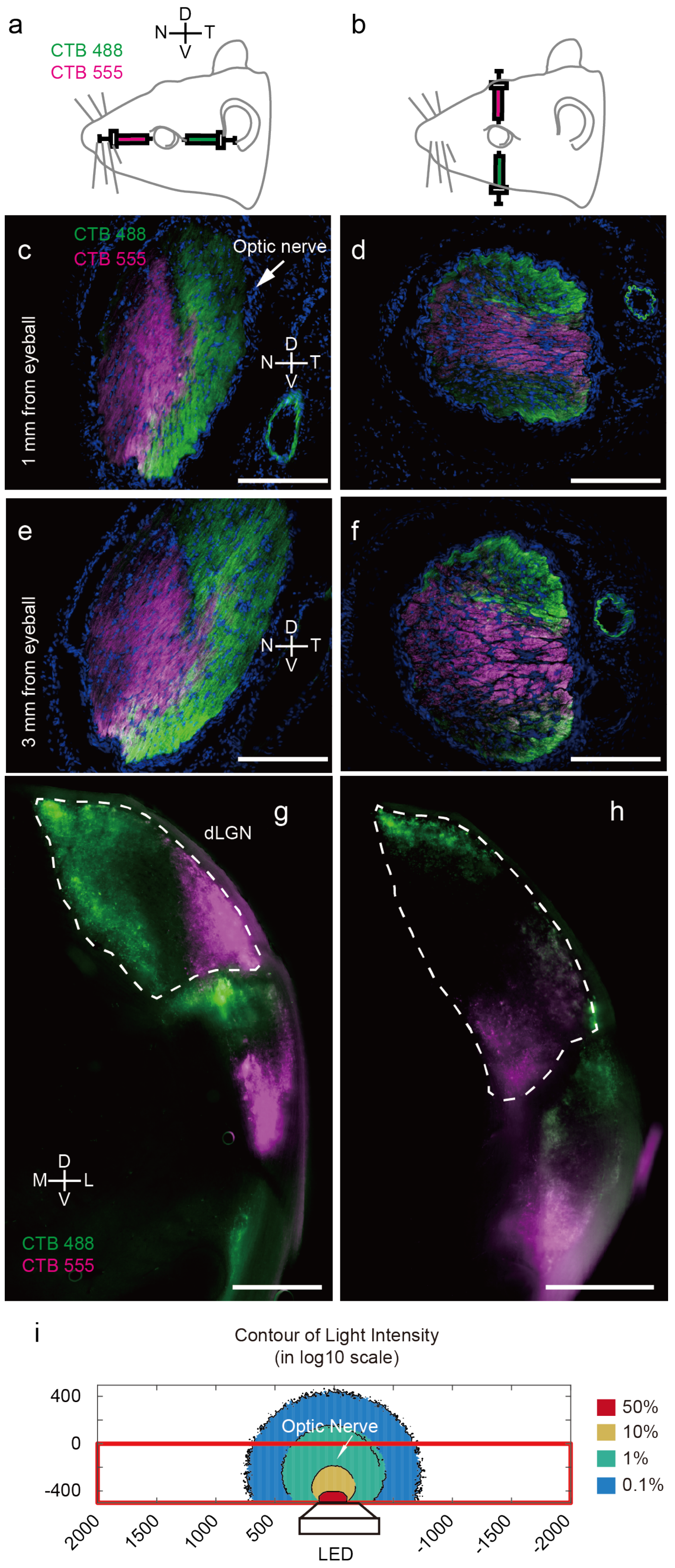
a
CTB 488
CTB 555
D
N
T
V
b
c
CTB 488
CTB 555
Optic nerve
1 mm from eyeball
d
e
3 mm from eyeball
f
g
dLGN
h
M
L
i
Contour of Light Intensity
(in log10 scale)
Optic Nerve
400
0
-400
2000
1500
1000
500
-1000
-1500
-2000
LED
50%
10%
1%
0.1%

141

Supplementary Figure 13. Topographic projection between optic nerve and lateral geniculate nucleus (LGN). (**a**, **b**) Intraocular injection of fluorescent dyes at different positions of the retina. CTB488 (green) and CTB555 (red) were intraocularly injected in a nasal-temporal or dorsal-ventral manner (700 nl per site, left eye). (**c**, **d**, **e**, **f**) Cross sections of optic nerve at 1 mm or 3 mm from the eye ball. Red, CTB555. Green, CTB488. Blue, DAPI. (**g**, **h**) Retinal axons in dorsal lateral geniculate nucleus (dLGN) and ventral lateral geniculate nucleus (vLGN). Red, CTB555. Green, CTB488. M, medial; L, lateral; D, dorsal; V, ventral. (**i**) Monte-Carlo simulation of light intensity distribution across and around the rat optic nerve on transverse section. Scale bars are 100 μm (**c-f**), 200 μm (**g-h**).

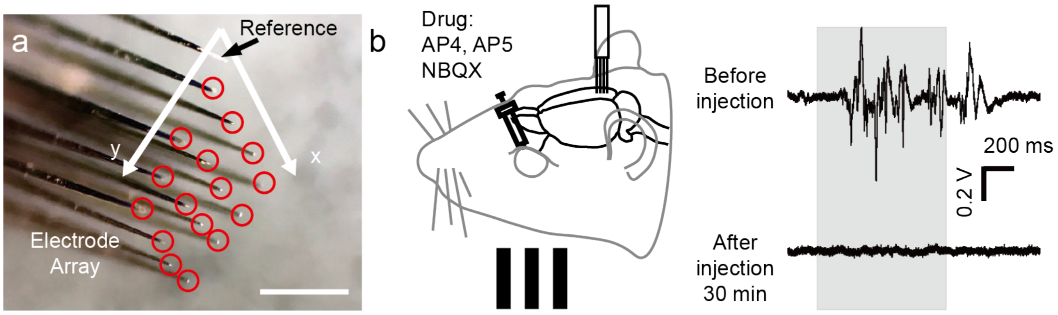

Supplementary Figure 14. Inhibition of responses to drifting gratings in V1 neurons using AP4, AP5 and NBQX. (**a**) Image of multichannel electrode array (MEA). (**b**) Schematics of the electrophysiology recordings with introvitreous injection of glutamertergic antagonist cocktail (left). Light responses in V1 before the injection and after injection 30 min (right). Shade area indicated the presentation of drifting gratings to the contralateral eye. Scale bar is 500 μm (**a**).

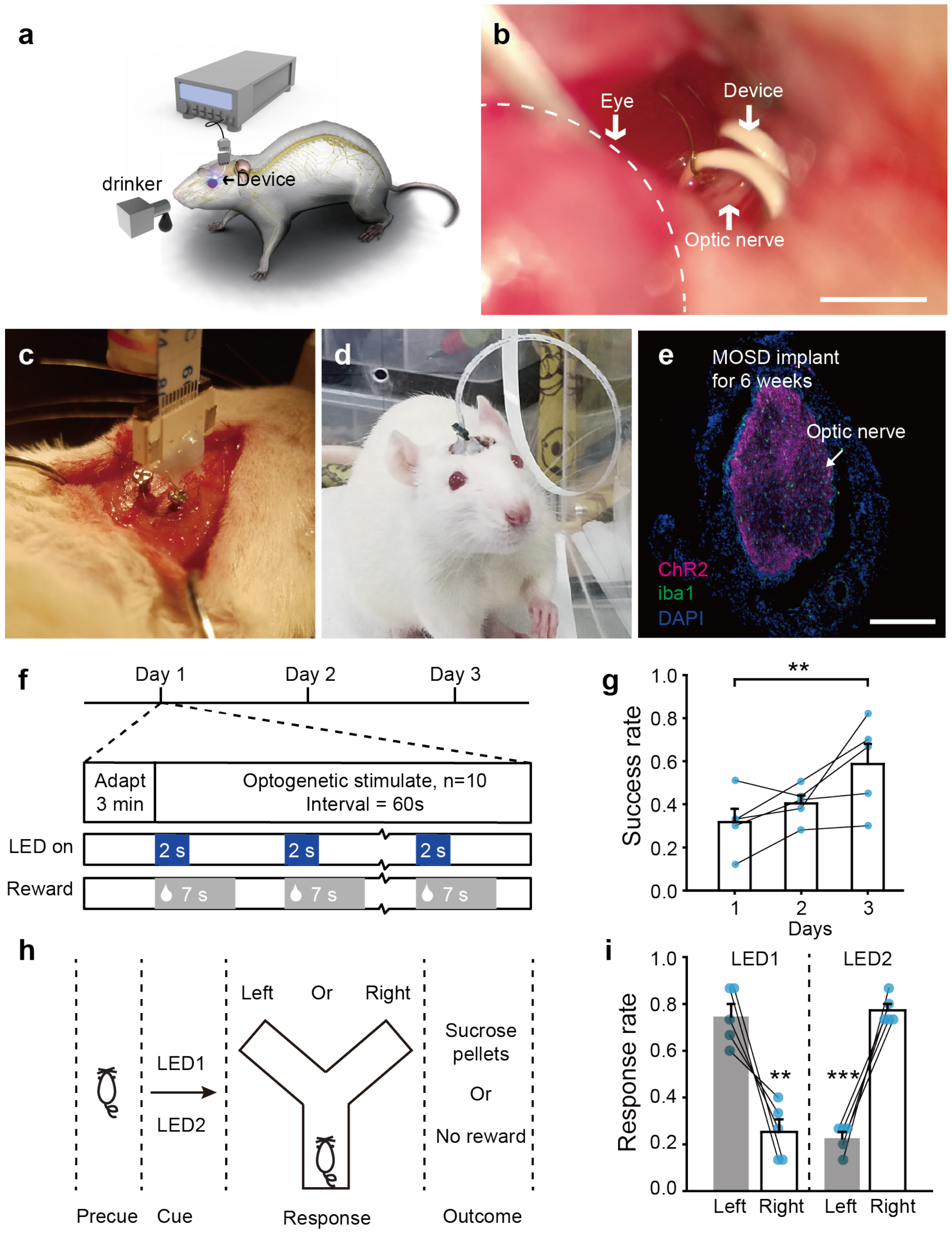

Supplementary Figure 15. Associated learning in freely moving rats implanted with MOSD. (**a**) Intraocular injection of AAV-hSyn-ChR2-mCherry (left). Schematic of the training chamber (right). (**b**) Image of the MOSD firmly implanted onto a rat's optic nerve. (**c**) Head-mounted flexible printed circuit adapter on the skull of the rat. (**d**) A postimplant-surgery rat wearing the head-mounted MOSD. (**e**) The immunohistochemistry from rat optic nerve with MOSD implanted over 3 weeks. (**f**) Behavioral training scheme.

After 3 min of habituation, each rat went through 10 training trials. In each 1-min trial, the mini-LED was turned on for 2 sec at 0.18 mW. The animal was allowed to lick water for 5 sec (0.05 mL water), followed by a 53-sec interval. (**g**) The ratio of successful water-licking trials to all trials in each training day. Data are represented as mean ± s.e.m. (n = 5 rats, 0.18 mW, LED1: 20 msec-on/ 20 msec-off, LED2: 500 msec-on/ 500 msec-off; One way repeated measures ANOVA with Tukey post hoc, **$P < 0.01$). (**h**) Schematics of 2-choice Y maze. The rats were cued with different mini-LEDs (0.18 mW, 20 msec-on/20 msec-off for mini-LED1 and 500 msec-on/500 msec-off for mini-LED2 for 10 sec) and rewarded with sucrose pellets if they chose the correct arm. (**i**) The response of rats in Y maze with distinct mini-LEDs (n = 5 rats, 0.18 mW, 2 sec, two way repeated measures ANOVA with Tukey post hoc). Data are represented as mean ± s.e.m.; **$P < 0.01$, ***$P < 0.001$. Scale bars are 1 mm (**b**), 200 μm (**e**).

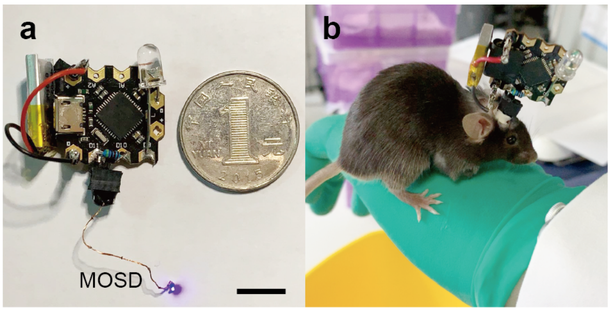

Supplementary Figure 16. Miniaturized stimulation headstage for MOSD. (**a**) MOSD and the miniaturized stimulation headstage. (**b**) A mouse implanted with MOSD and connected to the stimulation headstage. Scale bar is 1 cm (**a**).

Quantity	Symbol	Value	Unit	Source
Diameter of Sciatic Nerve	d	0.3	mm	Measured in this manuscript
Refractive Index of Sciatic Nerve	$n_{Sciatic\ Nerve}$	1.40	n.a.	Yaroslavsky, 2002[47]
Absorption Coefficient	μ_a	0.5	mm^{-1}	Yaroslavsky, 2002[47]
Reduced Scattering Coefficient	μ_s’	1.56	mm^{-1}	Jacques, 2013[46]
Anisotropy	g	0.90	n.a.	Jacques, 2013[46]

Supplementary Table 1. Parameters of optical properties for mice sciatic nerve and C7 nerve.

Mouse sciatic nerve		Light intensity [mW mm^{-2}]		
Voltage (V)	light Power (mW)	At the mini-LED	Middle	Far end
2.3	0.00003	0.0013	0.00059	0.00020
2.4	0.00007	0.0031	0.0014	0.00046
2.5	0.0012	0.052	0.023	0.0079
2.6	0.026	1.14	0.51	0.17
2.7	0.18	7.99	3.58	1.21
2.8	0.57	24.9	11.2	3.78
2.9	1.31	57.2	25.6	8.67
3	4.55	198.1	88.8	30.0
3.1	7.67	334.4	149.8	50.7
3.2	13.3	579.6	259.7	87.9
3.3	20.4	889.0	398.3	134.8
2.45	0.3	13.1	5.86	1.98

Supplementary Table 2. Monte Carlo simulation of light intensities at the mini-LED light-emitting surface, middle and far end of the cross section of the mice sciatic nerve.

Mouse C7		Light intensity [mW mm^{-2}]		
Voltage (V)	light Power (mW)	At the mini-LED	Middle	Far end
2.3	0.00003	0.0013	0.00089	0.00039
2.4	0.00007	0.0030	0.0021	0.00092
2.5	0.0012	0.05	0.035	0.015
2.6	0.026	1.14	0.77	0.34
2.7	0.18	7.99	5.41	2.40
2.8	0.57	24.9	16.9	7.49
2.9	1.31	57.2	38.7	17.2
3	4.55	198.1	134.2	59.5
3.1	7.67	334.4	226.6	100.5
3.2	13.3	579.6	392.6	174.2
3.3	20.4	889.0	602.2	267.2
2.45	0.3	13.1	8.86	3.93

Supplementary Table 3. Monte Carlo simulation of light intensities at the mini-LED light-emitting surface, middle and far end of the cross section of the mice C7 nerve.

Quantity	Symbol	Value	Unit	Source
Diameter of Optic Nerve	d	0.5	mm	Hughes, 1977[44]
Refractive Index of Optic Nerve	$n_{Optic\ Nerve}$	1.40	n.a.	Yaroslavsky, 2002[47]
Absorption Coefficient	μ_a	0.5	mm^{-1}	Yaroslavsky, 2002[47]
Reduced Scattering Coefficient	μ_s'	2.59	mm^{-1}	Jacques, 2013[46]
Anisotropy	g	0.90	n.a.	Jacques, 2013[46]

Supplementary Table 4. Parameters of optical properties for rat optic nerve.

Rat optic nerve		Light intensity [mW mm^{-2}]		
Voltage (V)	light Power (mW)	At the mini-LED	Middle	Far end
2.3	0.00003	0.0014	0.00025	0.000047
2.4	0.00007	0.0032	0.00058	0.00011
2.5	0.0012	0.06	0.0099	0.0019
2.6	0.026	1.21	0.22	0.041
2.7	0.18	8.47	1.51	0.29
2.8	0.57	26.4	4.72	0.89
2.9	1.31	60.6	10.8	2.05
3	4.55	209.9	37.5	7.09
3.1	7.67	354.3	63.3	12.0
3.2	13.3	614.0	109.7	20.7
3.3	20.4	941.8	168.3	31.8
2.45	0.3	13.9	2.48	0.47

Supplementary Table 5. Monte Carlo simulation of light intensities at the mini-LED light-emitting surface, middle and far end of the cross section of the rat optic nerve.

Figures	Voltge	Device	Power	Pulse width
Fig. 2g	2.5V	mini-LED	0.0012 mW	Continuously-on
Fig. 2j	2.3 V - 3.5 V	mini-LED	0.00003 mW - 33.1 mW	20 msec-on/ 2 sec-off 1 sec-on/ 4 sec-off
Fig. 3e	2.6V	mini-LED	0.026 mW	20 msec-on/ 2 sec-off
Fig. 3f	3.3V	mini-LED	20. 4 mW	20 msec-on/ 2 sec-off
Fig. 3g	2.9 - 3.3V	mini-LED	1.31mW - 20. 4 mW	20 msec-on/ 2 sec-off
Fig. 3h	3V	mini-LED	4.55 mW	20 msec-on/ 2 sec-off
Fig. 4d	3.3 V	mini-LED	20.4 mW	20 msec-on/ 2 sec-off
Fig. 4g	3.3 V	mini-LED	81. 6mW	20 msec-on/ 2 sec-off
Fig. 5i	3.3 V	mini-LED	20. 4 mW	20 msec-on/ 2 sec-off
Fig. 5j	2.3 V - 3.3 V	mini-LED	0.00003 mW - 20. 4 mW	20 msec-on/ 2 sec-off
Fig. 5k-p	2.6 V - 3.2 V	mini-LED	0.026mW – 13.30 mW	20 msec-on/ 2 sec-off
Fig. 6c	3.3 V	mini-LED	20. 4 mW	20 msec-on/ 2 sec-off
Fig. 6d	2.9 - 3.3 V	mini-LED	1.31mW - 20. 4 mW	20 msec-on/ 2 sec-off
Fig. 6f	3.3 V	mini-LED	81. 6 mW	20 msec-on/ 2 sec-off
Fig. 7k	3.3 V	mini-LED	81. 6 mW	20 msec-on/ 2 sec-off
Supplementary Figure 5a	2.5 V	mini-LED	0.0012 mW	4 hour
Supplementary Figure 5b	2.3-3.5V	mini-LED	0.00003 mW -33.1 mW	20 msec-on/ 2 sec-off 1 sec-on/ 4 sec-off
Supplementary Figure 9a	2.6 - 3.3 V	mini-LED	0.026 mW- 20. 4 mW	20 msec-on/ 2 sec-off
Supplementary Figure 10a	2.6 - 3.3 V	mini-LED	0.026 mW- 20. 4 mW	20 msec-on/ 2 sec-off
Supplementary Figure 11a	2.9V	mini-LED	1.31mW	20-msec-on/20-msec-off for mini-LED1 and 500-msec-on/500-msec-off for mini-LED2 for 10 sec
Supplementary Figure 11b, c	2.3 V - 3.3 V	mini-LED	0.00003 mW - 20. 4 mW	20 msec-on/ 2 sec-off
Supplementa	2.3 V - 2.7	mini-LED	0.00003 mW - 0.18 mW	1 sec-on/ 4 sec-off

ry Figure 12k	V			
Supplementary Figure 12l	2.7 V	mini-LED	0.18 mW	1 sec-on/ 4 sec-off
Supplementary Figure 12m	2.3 V	mini-LED	0.00003 mW	1 sec-on/ 4 sec-off
Supplementary Figure 12n	2.3-2.5V	mini-LED	0.00003 mW and 0.0012 mW	1 sec-on/ 4 sec-off
Supplementary Figure 12o	2.4 V and 2.5 V	mini-LED	0.00003 mW and 0.0012 mW	1 sec-on/ 4 sec-off
Supplementary Figure 15f, g	2.7 V	mini-LED	0.18 mW	LED1: 2 sec-on/ 60 sec-off
Supplementary Figure 15h, i	2.7V	mini-LED	0.18 mW	20-msec-on/20-msec-off for mini-LED1 and 500-msec-on/500-msec-off for mini-LED2 for 10 sec

Supplementary Table 6. Stimulation parameters used in the manuscript.

Figures		P value	n	Statistical analysis
Fig. 3g	TA	0.012	8（mice）	Paired t test
	GN	0.025	8（mice）	Wilcoxon Signed Rank Test
Fig. 3h	ES	0.069	3（mice）	Paired t test
	SS	0.917	5（mice）	Paired t test
Fig. 4d		<0.001	5（mice）	Paired t test
Fig. 4g	ES		3（mice）	
	SS		5（mice）	
Fig. 5k	Col 1 vs Col 4	0.001	4（trials）	Friedman’s ANOVA on Ranks with Tukey post hoc
	Col 1 vs Col 5	0.034		Friedman’s ANOVA on Ranks with Tukey post hoc
	Col 1 vs Col 2	0.451		Friedman’s ANOVA on Ranks with Tukey post hoc
	Col 1 vs Col 3	0.754		Friedman’s ANOVA on Ranks with Tukey post hoc
	Col 3 vs	0.065		Friedman’s ANOVA on Ranks with

	Col 4			Tukey post hoc
	Col 3 vs Col 5	0.451		Friedman's ANOVA on Ranks with Tukey post hoc
	Col 3 vs Col 2	0.989		Friedman's ANOVA on Ranks with Tukey post hoc
	Col 2 vs Col 4	0.199		Friedman's ANOVA on Ranks with Tukey post hoc
	Col 2 vs Col 5	0.754		Friedman's ANOVA on Ranks with Tukey post hoc
	Col 5 vs Col 4	0.875		Friedman's ANOVA on Ranks with Tukey post hoc
Fig. 5l	Col 2 vs Col 4	0.001	4（trials）	Friedman's ANOVA on Ranks with Tukey post hoc
	Col 2 vs Col 5	0.118		Friedman's ANOVA on Ranks with Tukey post hoc
	Col 2 vs Col 1	0.199		Friedman's ANOVA on Ranks with Tukey post hoc
	Col 2 vs Col 3	0.754		Friedman's ANOVA on Ranks with Tukey post hoc
	Col 3 vs Col 4	0.065		Friedman's ANOVA on Ranks with Tukey post hoc
	Col 3 vs Col 5	0.754		Friedman's ANOVA on Ranks with Tukey post hoc
	Col 3 vs Col 1	0.875		Friedman's ANOVA on Ranks with Tukey post hoc
	Col 1 vs Col 4	0.451		Friedman's ANOVA on Ranks with Tukey post hoc
	Col 1 vs Col 5	0.999		Friedman's ANOVA on Ranks with Tukey post hoc
	Col 5 vs Col 4	0.605		Friedman's ANOVA on Ranks with Tukey post hoc
Fig. 5m	Col 3 vs. Col 4	<0.001	4（trials）	One way ANOVA with Tukey post hoc
	Col 3 vs. Col 2	<0.001		One way ANOVA with Tukey post hoc
	Col 3 vs. Col 5	<0.001		One way ANOVA with Tukey post hoc
	Col 3 vs. Col 1	<0.001		One way ANOVA with Tukey post hoc
	Col 1 vs. Col 4	<0.001		One way ANOVA with Tukey post hoc
	Col 1 vs. Col 2	0.001		One way ANOVA with Tukey post hoc

	Col 1 vs. Col 5	0.141		One way ANOVA with Tukey post hoc
	Col 5 vs. Col 4	0.007		One way ANOVA with Tukey post hoc
	Col 5 vs. Col 2	0.122		One way ANOVA with Tukey post hoc
	Col 2 vs. Col 4	0.566		One way ANOVA with Tukey post hoc
Fig. 5n	Col 4 vs. Col 5	<0.001	4（trials）	One way ANOVA with Tukey post hoc
	Col 4 vs. Col 2	<0.001		One way ANOVA with Tukey post hoc
	Col 4 vs. Col 1	<0.001		One way ANOVA with Tukey post hoc
	Col 4 vs. Col 3	<0.001		One way ANOVA with Tukey post hoc
	Col 3 vs. Col 5	<0.001		One way ANOVA with Tukey post hoc
	Col 3 vs. Col 2	<0.001		One way ANOVA with Tukey post hoc
	Col 3 vs. Col 1	0.88		One way ANOVA with Tukey post hoc
	Col 1 vs. Col 5	<0.001		One way ANOVA with Tukey post hoc
	Col 1 vs. Col 2	<0.001		One way ANOVA with Tukey post hoc
	Col 2 vs. Col 5	0.096		One way ANOVA with Tukey post hoc
Fig. 5o	Col 5 vs Col 3	0.007	4（trials）	Friedman's ANOVA on Ranks with Tukey post hoc
	Col 5 vs Col 1	0.034		Friedman's ANOVA on Ranks with Tukey post hoc
	Col 5 vs Col 4	0.451		Friedman's ANOVA on Ranks with Tukey post hoc
	Col 5 vs Col 2	0.989		Friedman's ANOVA on Ranks with Tukey post hoc
	Col 2 vs Col 3	0.034		Friedman's ANOVA on Ranks with Tukey post hoc
	Col 2 vs Col 1	0.118		Friedman's ANOVA on Ranks with Tukey post hoc
	Col 2 vs Col 4	0.754		Friedman's ANOVA on Ranks with Tukey post hoc
	Col 4 vs Col 3	0.451		Friedman's ANOVA on Ranks with Tukey post hoc

	Col 4 vs Col 1	0.754		Friedman's ANOVA on Ranks with Tukey post hoc
	Col 1 vs Col 3	0.989		Friedman's ANOVA on Ranks with Tukey post hoc
Fig. 5p	Col 5 vs Col 1	0.005	4（trials）	Friedman's ANOVA on Ranks with Tukey post hoc
	Col 5 vs Col 3	0.102		Friedman's ANOVA on Ranks with Tukey post hoc
	Col 5 vs Col 4	0.118		Friedman's ANOVA on Ranks with Tukey post hoc
	Col 5 vs Col 2	0.719		Friedman's ANOVA on Ranks with Tukey post hoc
	Col 2 vs Col 1	0.175		Friedman's ANOVA on Ranks with Tukey post hoc
	Col 2 vs Col 3	0.754		Friedman's ANOVA on Ranks with Tukey post hoc
	Col 2 vs Col 4	0.788		Friedman's ANOVA on Ranks with Tukey post hoc
	Col 4 vs Col 1	0.819		Friedman's ANOVA on Ranks with Tukey post hoc
	Col 4 vs Col 3	1		Friedman's ANOVA on Ranks with Tukey post hoc
	Col 3 vs Col 1	0.848		Friedman's ANOVA on Ranks with Tukey post hoc
Fig. 6d shoulder adduction	Col 2 vs Col 1	<0.001	14（mice）	Friedman's RM ANOVA on Ranks with Tukey post hoc
	Col 2 vs Col 4	0.227		Friedman's RM ANOVA on Ranks with Tukey post hoc
	Col 2 vs Col 3	0.552		Friedman's RM ANOVA on Ranks with Tukey post hoc
	Col 3 vs Col 1	0.001		Friedman's RM ANOVA on Ranks with Tukey post hoc
	Col 3 vs Col 4	0.936		Friedman's RM ANOVA on Ranks with Tukey post hoc
	Col 4 vs Col 1	0.011		Friedman's RM ANOVA on Ranks with Tukey post hoc
Fig. 6d elbow extension	Col 2 vs Col 1	<0.001	14（mice）	Friedman's RM ANOVA on Ranks with Tukey post hoc
	Col 2 vs Col 3	<0.001		Friedman's RM ANOVA on Ranks with Tukey post hoc
	Col 2 vs Col 4	0.001		Friedman's RM ANOVA on Ranks with Tukey post hoc

	Col 4 vs Col 1	0.912		Friedman's RM ANOVA on Ranks with Tukey post hoc
	Col 4 vs Col 3	0.999		Friedman's RM ANOVA on Ranks with Tukey post hoc
	Col 3 vs Col 1	0.956		Friedman's RM ANOVA on Ranks with Tukey post hoc
Fig. 6d wrist extension	Col 3 vs. Col 4	<0.001	7（mice）	One way RM ANOVA with Tukey post hoc
	Col 3 vs. Col 1	<0.001		One way RM ANOVA with Tukey post hoc
	Col 3 vs. Col 2	<0.001		One way RM ANOVA with Tukey post hoc
	Col 2 vs. Col 4	0.372		One way RM ANOVA with Tukey post hoc
	Col 2 vs. Col 1	1		One way RM ANOVA with Tukey post hoc
	Col 1 vs. Col 4	0.378		One way RM ANOVA with Tukey post hoc
Fig. 6d wrist flexion	Col 4 vs Col 3	<0.001	12（mice）	Friedman's RM ANOVA on Ranks with Tukey post hoc
	Col 4 vs col 1	0.045		Friedman's RM ANOVA on Ranks with Tukey post hoc
	Col 4 vs Col 2	0.685		Friedman's RM ANOVA on Ranks with Tukey post hoc
	Col 2 vs Col 3	0.001		Friedman's RM ANOVA on Ranks with Tukey post hoc
	Col 2 vs Col 1	0.436		Friedman's RM ANOVA on Ranks with Tukey post hoc
	Col 1 vs Col 3	0.119		Friedman's RM ANOVA on Ranks with Tukey post hoc
Fig. 6d finger extension	Col 4 vs Col 3	<0.001	10（mice）	Friedman's RM ANOVA on Ranks with Tukey post hoc
	Col 4 vs Col 1	0.003		Friedman's RM ANOVA on Ranks with Tukey post hoc
	Col 4 vs Col 2	0.046		Friedman's RM ANOVA on Ranks with Tukey post hoc
	Col 2 vs Col 3	0.307		Friedman's RM ANOVA on Ranks with Tukey post hoc
	Col 2 vs Col 1	0.822		Friedman's RM ANOVA on Ranks with Tukey post hoc
	Col 1 vs	0.822		Friedman's RM ANOVA on Ranks with

	Col 3			Tukey post hoc
Fig. 6e	ES		5（mice）	/
Fig. 6f	SS		3（mice）	/
Fig. 7d		0.115	Implant:<br>4（mice）；<br>No Implant<br>6（mice）	Unpaired t test
Fig. 7f		0.642	Implant:<br>4（mice）；<br>No Implant:<br>6（mice）	Unpaired t test
Fig. S9	SS		5（mice）	
	ES		3（mice）	
Fig. S10	SS		7（mice）	
	ES		3（mice）	
Fig. S12o		0.033	7（rats）	Paired t test
Fig. S15g	Col 1 vs Col 3	0.008	5（rats）	One way repeated measures ANOVA with Tukey post hoc
Fig. S15i	Led1	0.001	5（rats）	Two way repeated measures ANOVA with Tukey post hoc
	Led2	<0.001	5（rats）	Two way repeated measures ANOVA with Tukey post hoc

Supplementary Table 7. Summary for statistical details in the manuscript.
