# Supplementary material for: A shape-memory and spiral light-emitting device for precise multisite stimulation of nerve bundles
Source: Nat Commun. 2019 Jun 26;10:2790. doi: 10.1038/s41467-019-10418-3 (PMC6594927; doi:10.1038/s41467-019-10418-3)
Supplement: Supplementary file 2 — Description of Additional Supplementary Files [file 41467_2019_10418_MOESM2_ESM.pdf]

Description of Additional Supplementary Files

File name: Supplementary Movie 1

Description: MOSD was placed onto the mice sciatic nerve and linearly elongated using a pair of tweezers with LED on.

File name: Supplementary Movie 2

Description: The MOSD was placed onto the mice sciatic nerve and linearly elongated with LED on 10 months after fabrication.

File name: Supplementary Movie 3

Description: Representative video of selective stimulation of the sciatic nerve by led1-2 of MOSD (light turned on at 20. 4 mW for 20 msec with 2-sec intervals).

File name: Supplementary Movie 4

Description: Representative video of ankle joint movement by electrical stimulation (0.7 mA, 0.2 msec).

File name: Supplementary Movie 5

Description: Representative video of ankle joint movement by singlesite optogenetic stimulation (81. 6 mW, 20 msec width-2sec interval).

File name: Supplementary Movie 6

Description: Representative video of upper limb movement elicited by different mini-LEDs of MOSD (20.4 mW, 20 msec width-2sec interval).

File name: Supplementary Movie 7

Description: Representative video of upper limb movement by electrical stimulation (0.8mA, 0.2 msec-on/ 1 sec-off).

File name: Supplementary Movie 8

Description: Representative video of upper limb movement by singlesite optogenetic stimulation (81.6 mW, 20 msec width-2sec interval).

File name: Supplementary Movie 9

Description: Representative video of upper limb movement elicited by LED 1-2 of MOSD (81. 6 mW, 20 msec width-2sec interval) 3 weeks after the C7 sever, anastomosis and MOSD implant surgery
