# Supplementary material for: A shape-memory and spiral light-emitting device for precise multisite stimulation of nerve bundles
Source: Nat Commun. 2019 Jun 26;10:2790. doi: 10.1038/s41467-019-10418-3 (PMC6594927; doi:10.1038/s41467-019-10418-3)
Supplement: Supplementary file 12 — Reporting Summary [file 41467_2019_10418_MOESM12_ESM.pdf]

nature research

Corresponding author(s): Jiayi Zhang, Wendong Xu, Huisheng Peng, NCOMMS_18-23745-A.

Last updated by author(s): Apr 30, 2019

Reporting Summary

Nature Research wishes to improve the reproducibility of the work that we publish. This form provides structure for consistency and transparency in reporting. For further information on Nature Research policies, see Authors & Referees and the Editorial Policy Checklist.

Statistics

For all statistical analyses, confirm that the following items are present in the figure legend, table legend, main text, or Methods section.

n/a	Confirmed	
☐	☒	The exact sample size (*n*) for each experimental group/condition, given as a discrete number and unit of measurement
☐	☒	A statement on whether measurements were taken from distinct samples or whether the same sample was measured repeatedly
☐	☒	The statistical test(s) used AND whether they are one- or two-sided<br>*Only common tests should be described solely by name; describe more complex techniques in the Methods section.*
☒	☐	A description of all covariates tested
☐	☒	A description of any assumptions or corrections, such as tests of normality and adjustment for multiple comparisons
☐	☒	A full description of the statistical parameters including central tendency (e.g. means) or other basic estimates (e.g. regression coefficient) AND variation (e.g. standard deviation) or associated estimates of uncertainty (e.g. confidence intervals)
☐	☒	For null hypothesis testing, the test statistic (e.g. F, t, r) with confidence intervals, effect sizes, degrees of freedom and P value noted<br>*Give P values as exact values whenever suitable.*
☒	☐	For Bayesian analysis, information on the choice of priors and Markov chain Monte Carlo settings
☒	☐	For hierarchical and complex designs, identification of the appropriate level for tests and full reporting of outcomes
☒	☐	Estimates of effect sizes (e.g. Cohen's d, Pearson's r), indicating how they were calculated

Our web collection on statistics for biologists contains articles on many of the points above.

Software and code

Policy information about availability of computer code

Data collection	shapr 3D was used in iPad pro to make 3d modeling;<br>KeyShot 7 was used to rendering the model;<br>Camera in iPhone XS Max was used to take the photo under stereo microscope and the surgical photo;<br>iMovie was used to edit the movie;<br>LabView was used to measure material performance;<br>Keithley Test Script Builder software(KEITHLEY) was used to control the MOSD through the power source system;<br>In vivo electrophysiology data was collected by CED Spike2 software;<br>Fluorescent images was collected by NIS-Elements BR;<br>Movies were collected by Camera in iPhone XS Max.
Data analysis	Image analysis: Python3, Adobe Photoshop CS6, Adobe Illustrator CC 2017;<br>Graphic illustration: Python3, Matlab 2016b, originPro8, Adobe illustrator CC 2017;<br>In vivo electrophysiology data analysis: CED Spike2, MATLAB R2016b, Python3;<br>Movement analysis: Python3, Adobe Photoshop CS6, Adobe Illustrator CC 2017.

For manuscripts utilizing custom algorithms or software that are central to the research but not yet described in published literature, software must be made available to editors/reviewers. We strongly encourage code deposition in a community repository (e.g. GitHub). See the Nature Research guidelines for submitting code & software for further information.

Data

Policy information about availability of data

All manuscripts must include a data availability statement. This statement should provide the following information, where applicable:

- Accession codes, unique identifiers, or web links for publicly available datasets
- A list of figures that have associated raw data
- A description of any restrictions on data availability

The data underlying Figures 3, 5 and Supplementary Figures 9-12 are provided in the following DOI: 10.12751/g-node.52349d. Rest of the relevant data is available from the corresponding author upon reasonable request.

Field-specific reporting

Please select the one below that is the best fit for your research. If you are not sure, read the appropriate sections before making your selection.

☒ Life sciences ☐ Behavioural & social sciences ☐ Ecological, evolutionary & environmental sciences

For a reference copy of the document with all sections, see nature.com/documents/nr-reporting-summary-flat.pdf

Life sciences study design

All studies must disclose on these points even when the disclosure is negative.

Sample size	No sample size calculations were performed. The sample size (n) of each experiment is provided in the corresponding figure captions in the paper and supplementary information. Sample sizes were chosen to support meaningful conclusions.
Data exclusions	No data were excluded from the analyses.
Replication	Animal experiments were performed on biological replicates following identical procedures to verify the reproducibility of the experimental findings.
Randomization	The mice used in experiments were randomly allocated into the positive and control groups.
Blinding	Investigators were not blinded to group allocation during data collection and analysis.

Reporting for specific materials, systems and methods

We require information from authors about some types of materials, experimental systems and methods used in many studies. Here, indicate whether each material, system or method listed is relevant to your study. If you are not sure if a list item applies to your research, read the appropriate section before selecting a response.

Materials & experimental systems

n/a	Involved in the study	
☐	☒	Antibodies
☒	☐	Eukaryotic cell lines
☒	☐	Palaeontology
☐	☒	Animals and other organisms
☒	☐	Human research participants
☒	☐	Clinical data

Methods

n/a	Involved in the study	
☒	☐	ChIP-seq
☒	☐	Flow cytometry
☒	☐	MRI-based neuroimaging

Antibodies

Antibodies used	The GFP antibody was purchased from Abcam Inc, UK. The DsRed antibody was purchased from clontech Inc, US. The BMP antibody was purchased from abcam, UK. The DAPI was purchased from sigma USA. The brn3a antibody was purchased from Santa Cruz Biotechnology, USA. The iba1 antibody was purchased from Wako, JP.
Validation	Validation details of the primary antibodies are available on the manufacturers' websites and the related references:GFP, Cat# GFP Cat# ab13970 antibody, https://www.abcam.com/gfp-antibody-ab13970.html, Ganley R P et al (pubmed id: 25972186); DsRed Polyclonal Antibody, Cat# 632496, https://www.takarabio.com/products/antibodies-and-elisa/fluorescent-protein-antibodies/red-fluorescent-protein-antibodies?catalog=632496, Steculorum S M et al (pubmed id: 27015310); BMP antibody, Cat# ab7349, https://www.abcam.cn/myelin-basic-protein-antibody-12-ab7349.html, Weider M et al. (pubmed id: 29500351); DAPI, Cat# D8417, https://www.sigmaaldrich.com/catalog/product/sigma/d8417?lang=zh&region=CN, Takahashi K et al (pubmed id: 16904174); brn3a antibody, Cat# sc-31984, https://www.scbt.com/scbt/product/brn-3a-antibody-c-20?

requestFrom=search, Diao Y et al (pubmed id: 28334242); iba1 antibody, Cat# 019-19741, http://www.wako-chem.co.jp/english/labchem/product/life/iba1_mono/index.htm, Ohsawa K et al (pubmed id: 10934045).

Animals and other organisms

Policy information about studies involving animals; ARRIVE guidelines recommended for reporting animal research

Laboratory animals	8-week old C57BL/6J mice were purchased from Shanghai Laboratory Animal Center, Chinese Academy of Sciences.<br>8-week old Thy1-ChR2-EYFP mice were purchased from the Jackson Laboratory.
Wild animals	This study do not involve wild animals.
Field-collected samples	No field-collected samples were used in the study.
Ethics oversight	Animal care and experiments were performed in accordance with the National Institutes of Health Guide for the Care and Use of Laboratory Animals and were approved by the Animal Care and Use Committee of Shanghai Medical College of Fudan University.

Note that full information on the approval of the study protocol must also be provided in the manuscript.
